# Supplementary figures and images for: Reactive oxygen species activate the Drosophila TNF receptor Wengen for damage-induced regeneration
Source: EMBO J. 2024 Jul 17;43(17):3604–26. doi: 10.1038/s44318-024-00155-9 (PMC11377715; doi:10.1038/s44318-024-00155-9)

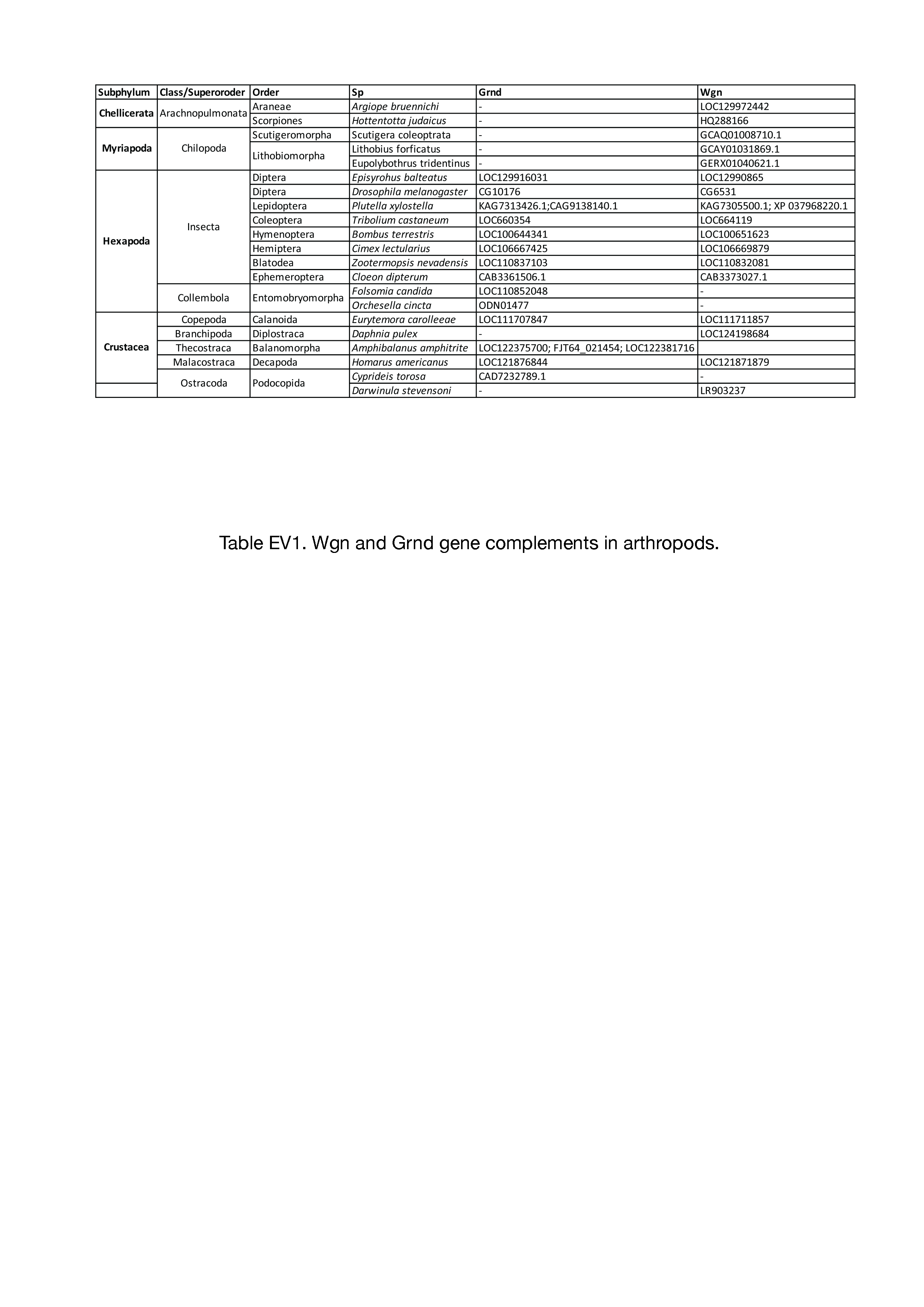

Supplement: Supplementary file 1 — Table EV1 [file 44318_2024_155_MOESM1_ESM.tif]

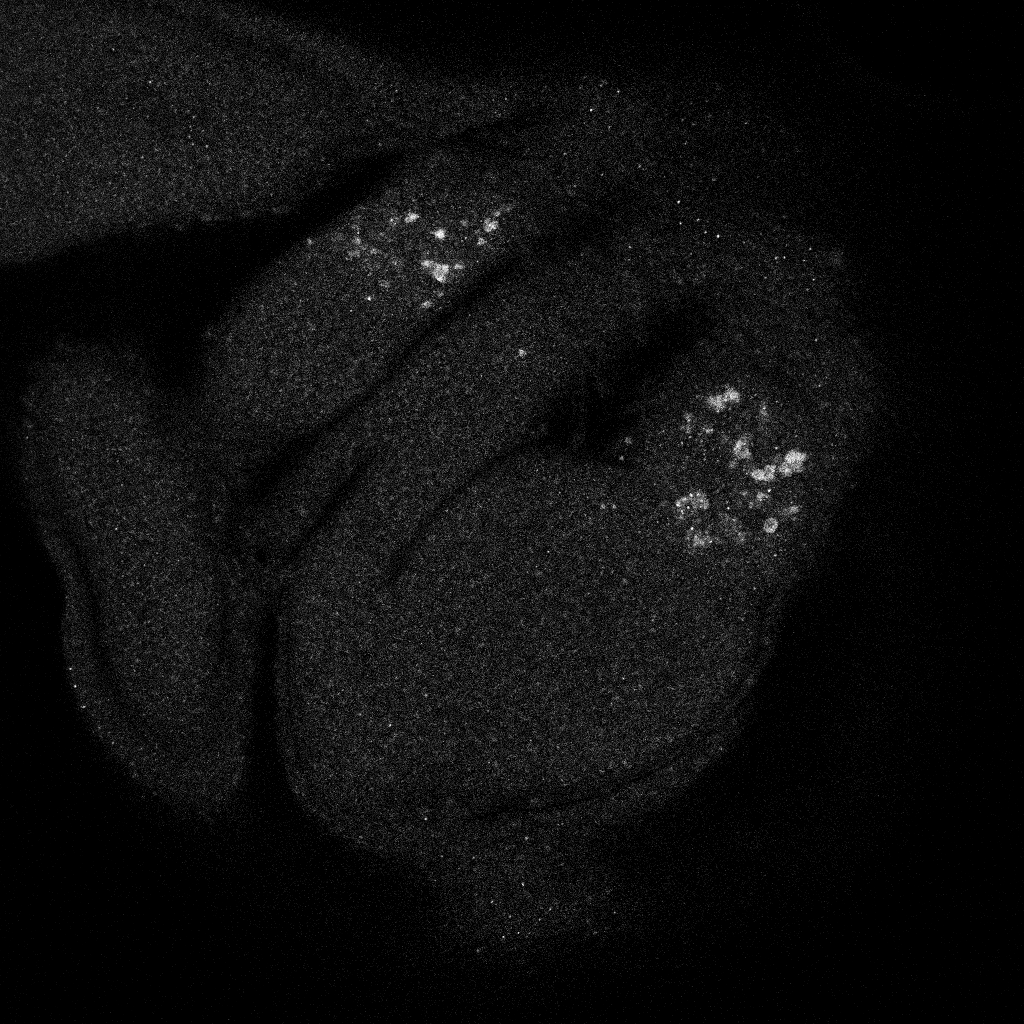

Supplement: Supplementary file 3 — Source data Fig. 1 [file 44318_2024_155_MOESM3_ESM.zip › Fig 1/Fig 1G/1G stack.tif]

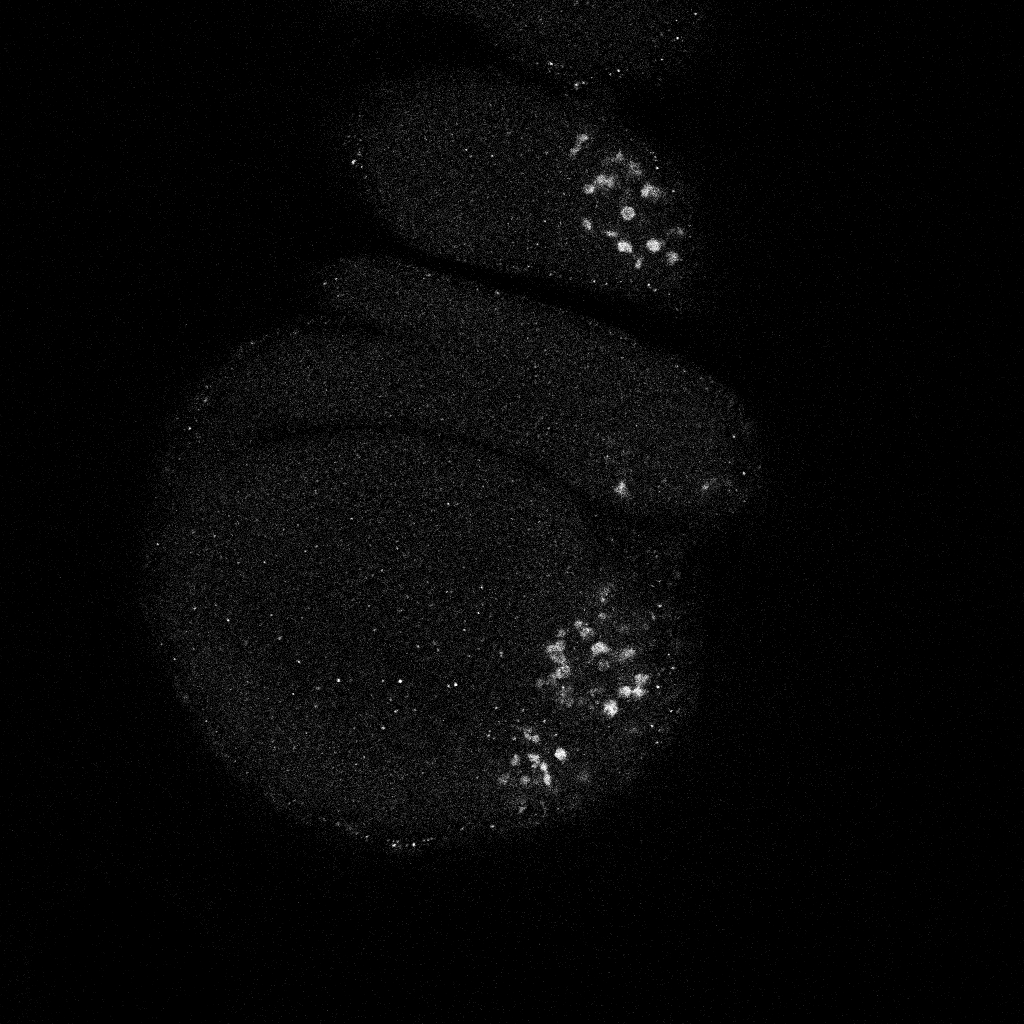

Supplement: Supplementary file 3 — Source data Fig. 1 [file 44318_2024_155_MOESM3_ESM.zip › Fig 1/Fig 1F/1F stack.tif]

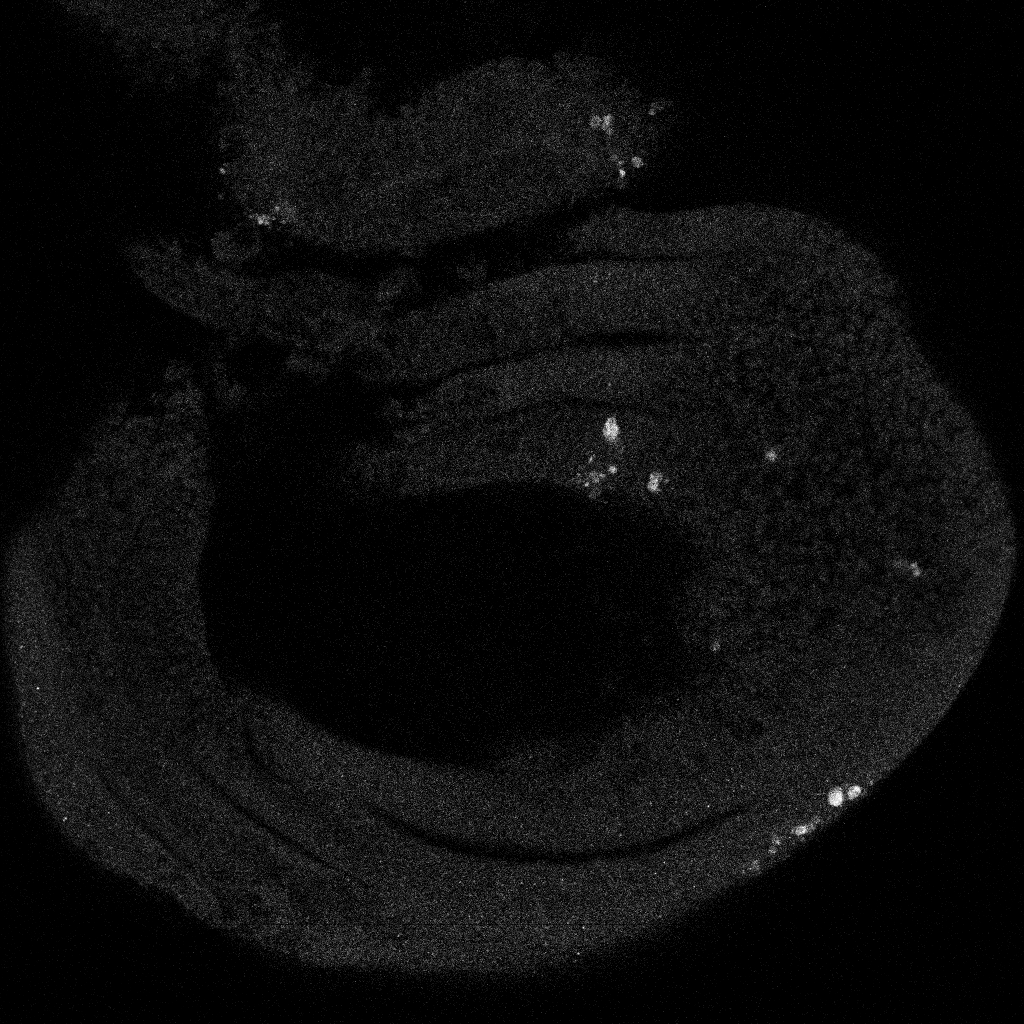

Supplement: Supplementary file 3 — Source data Fig. 1 [file 44318_2024_155_MOESM3_ESM.zip › Fig 1/Fig 1C/1C stack.tif]

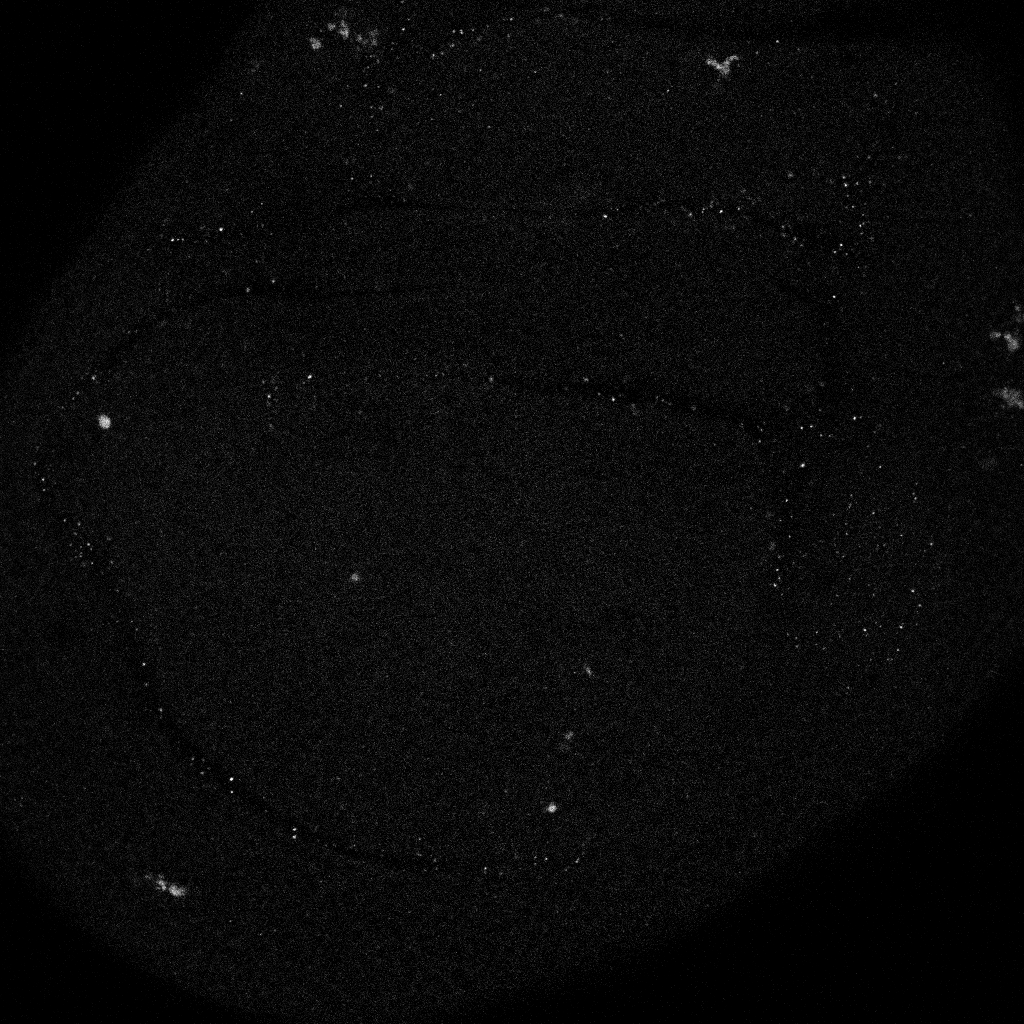

Supplement: Supplementary file 3 — Source data Fig. 1 [file 44318_2024_155_MOESM3_ESM.zip › Fig 1/Fig 1B/1B stack.tif]

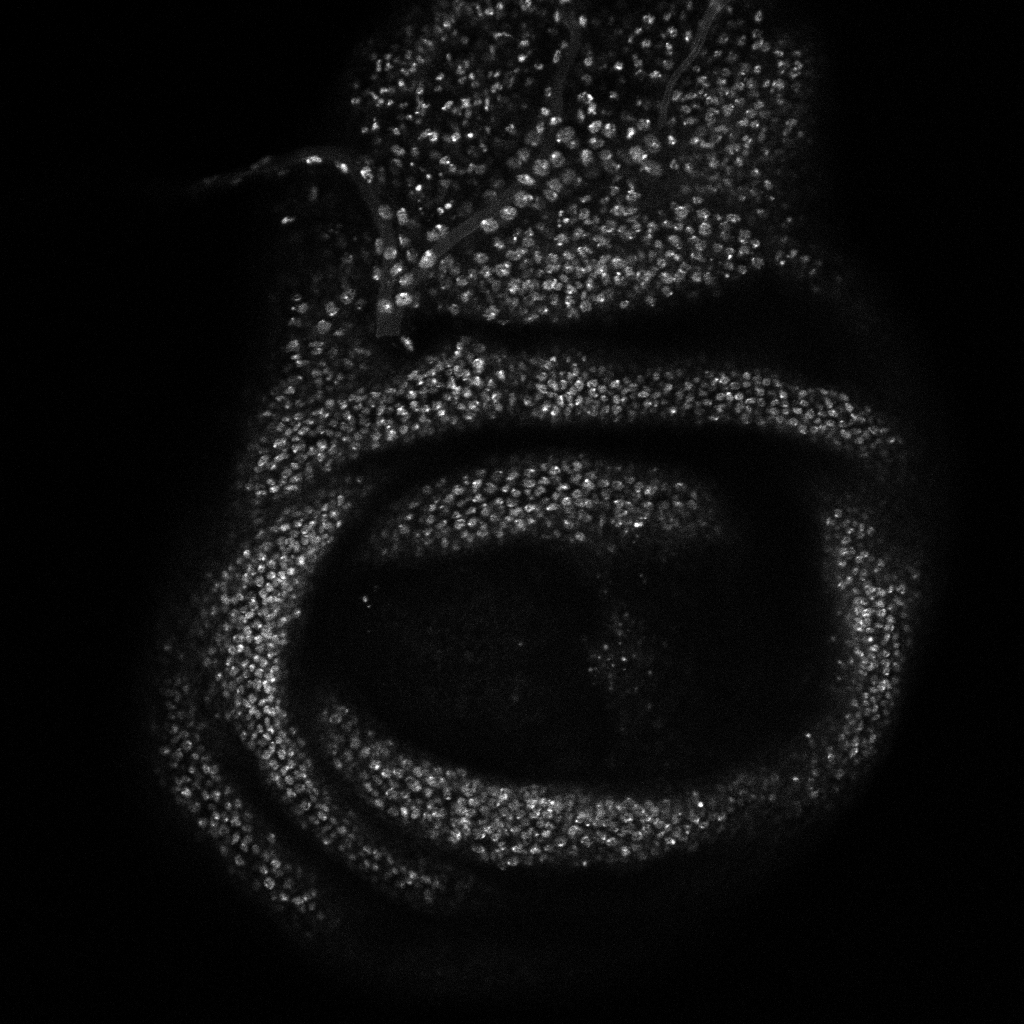

Supplement: Supplementary file 3 — Source data Fig. 1 [file 44318_2024_155_MOESM3_ESM.zip › Fig 1/Fig 1E/1E stack.tif]

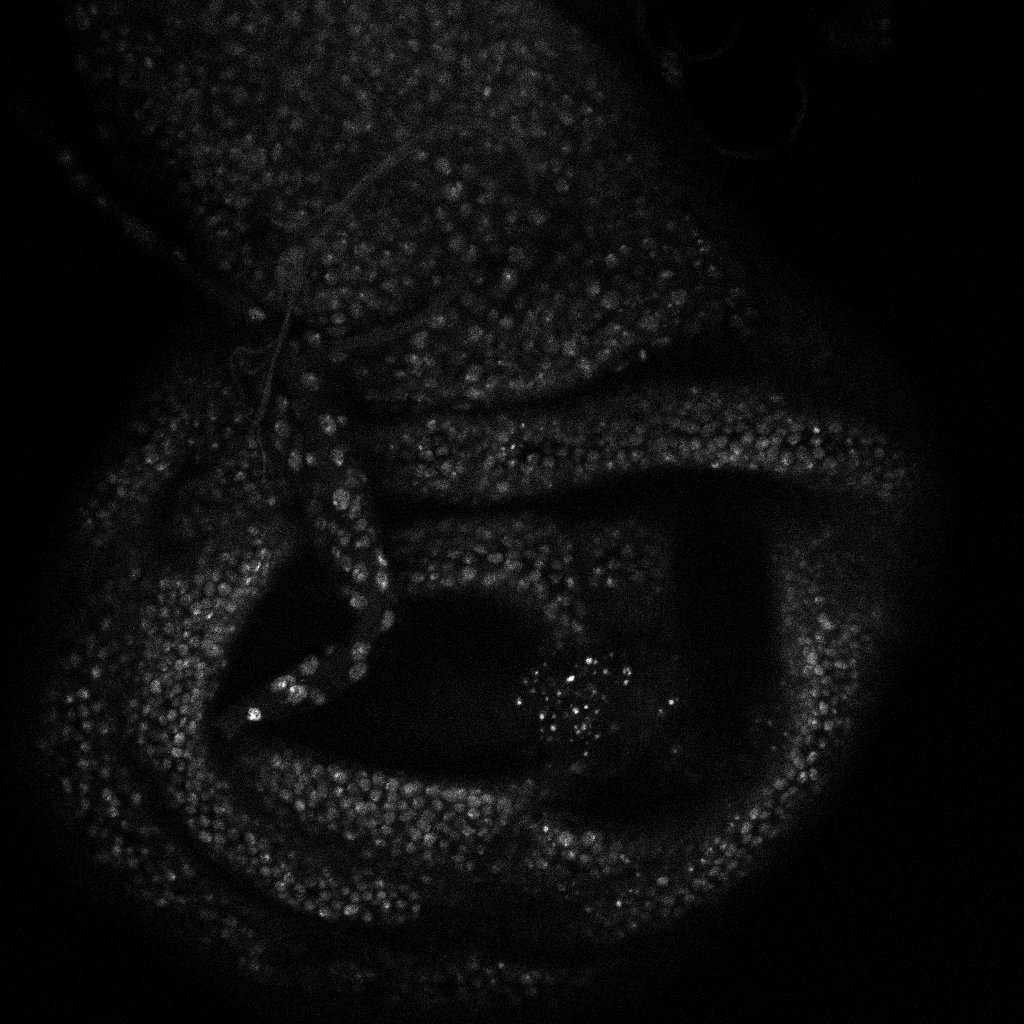

Supplement: Supplementary file 4 — Source data Fig. 2 [file 44318_2024_155_MOESM4_ESM.zip › Fig 2/Fig 2A/2A stack.tif]

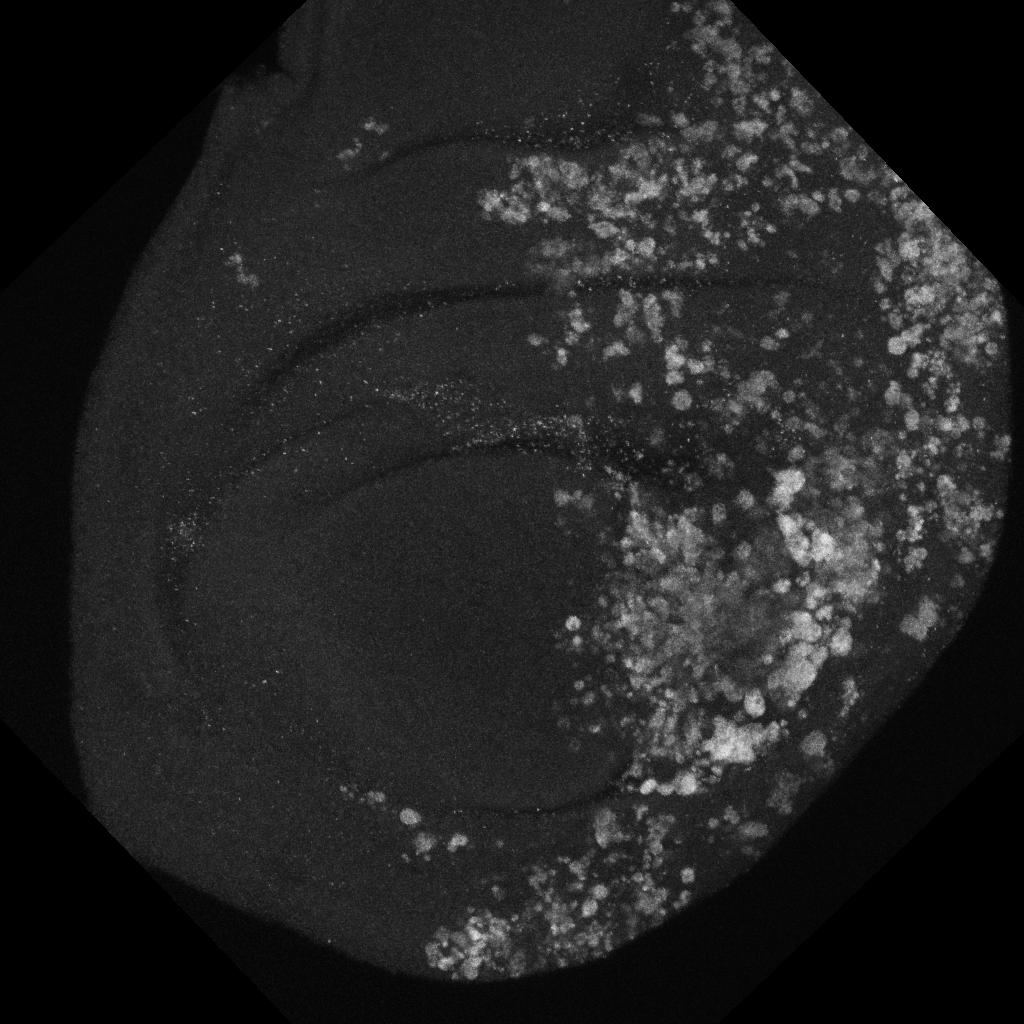

Supplement: Supplementary file 4 — Source data Fig. 2 [file 44318_2024_155_MOESM4_ESM.zip › Fig 2/Fig 2C/2C stack.tif]

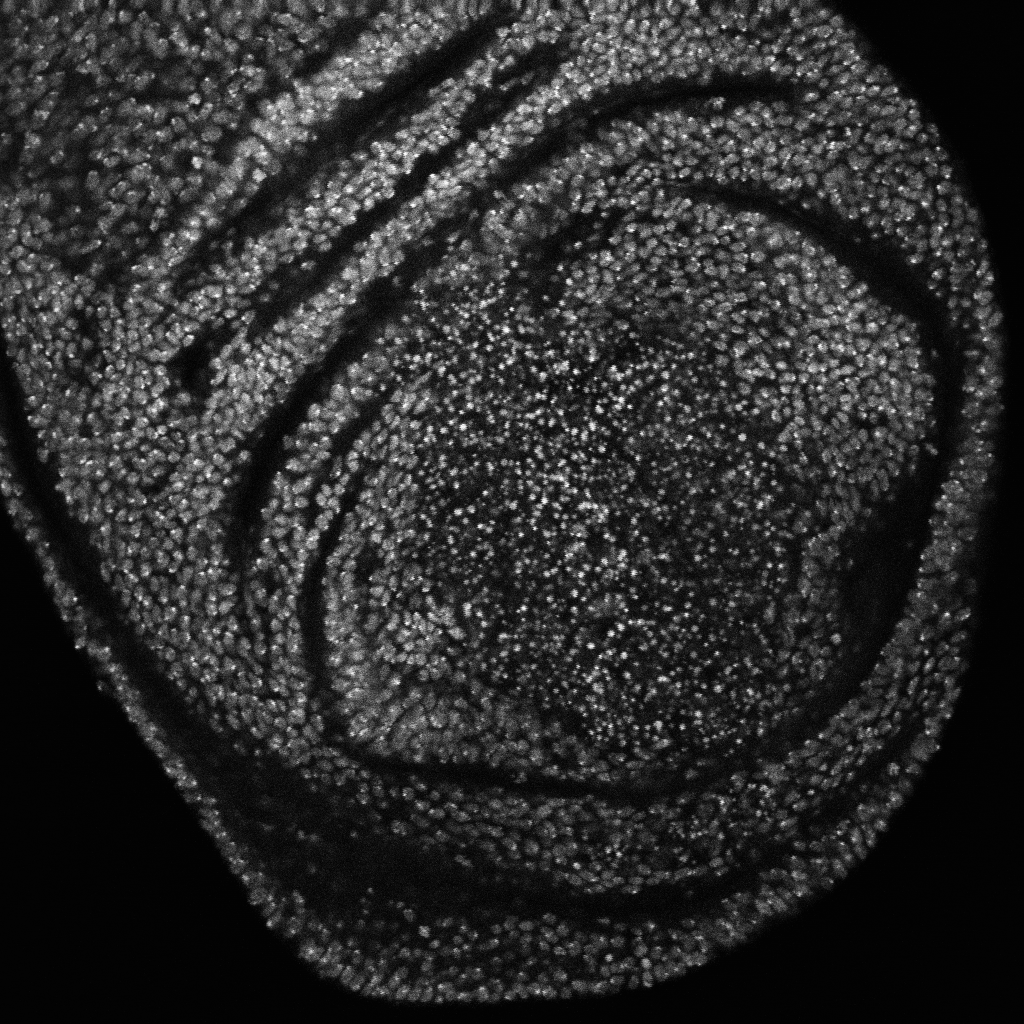

Supplement: Supplementary file 7 — Source data Fig. 5 [file 44318_2024_155_MOESM7_ESM.zip › Fig 5/Fig 5F/5F nuclei.tif]

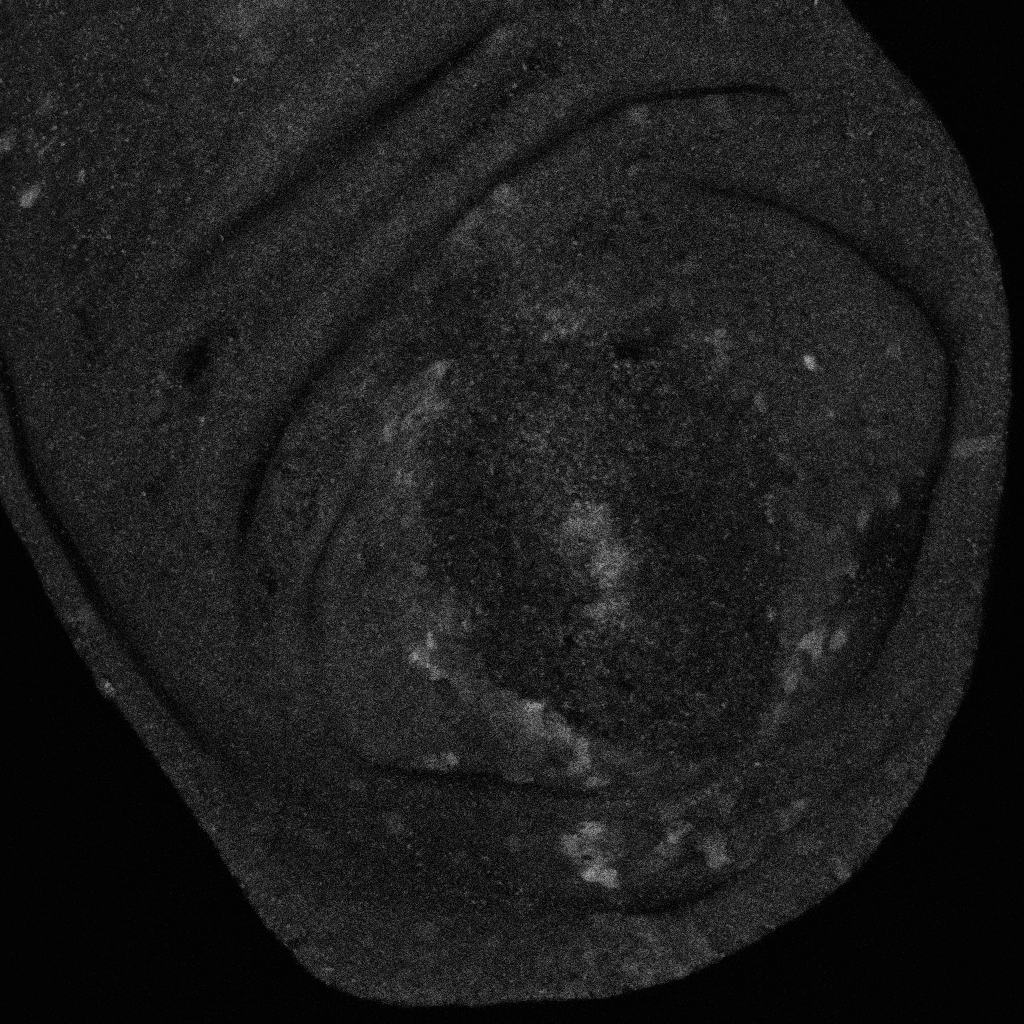

Supplement: Supplementary file 7 — Source data Fig. 5 [file 44318_2024_155_MOESM7_ESM.zip › Fig 5/Fig 5F/5F P-p38.tif]

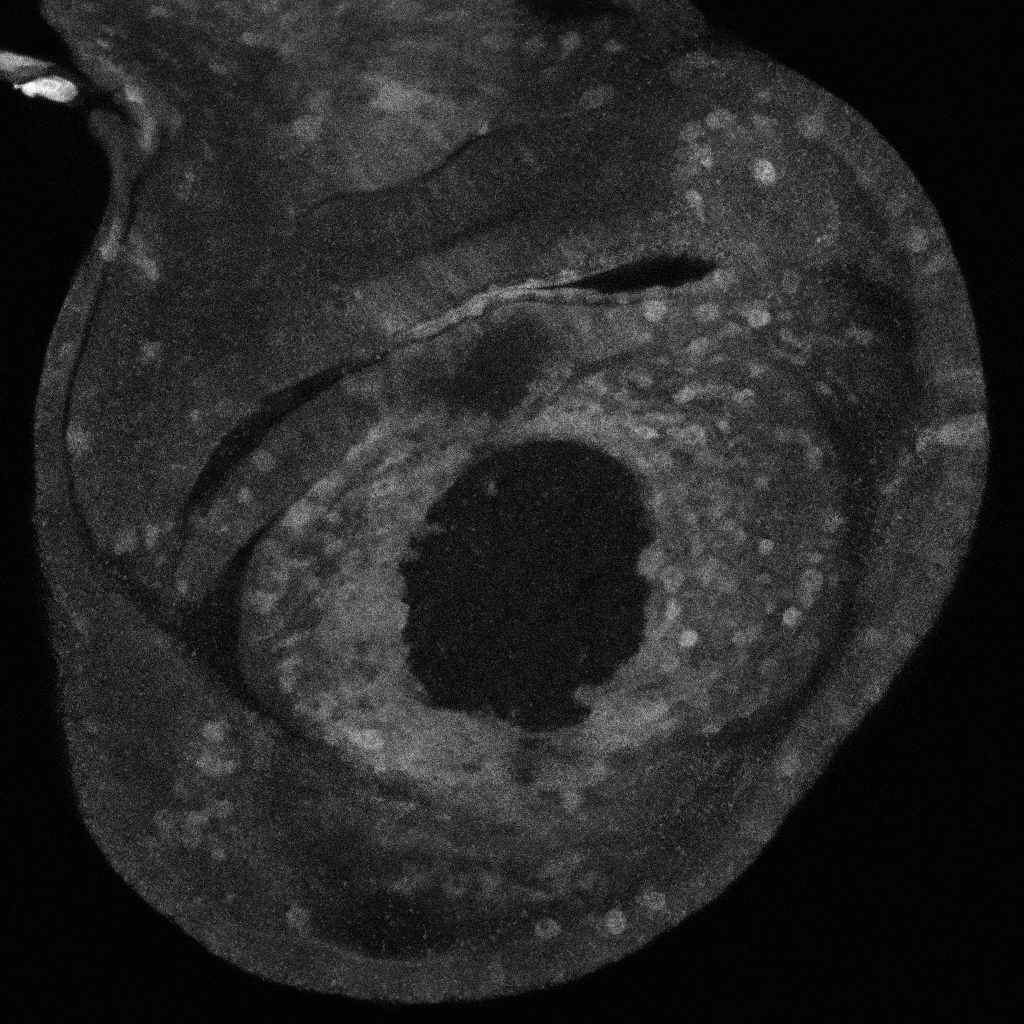

Supplement: Supplementary file 7 — Source data Fig. 5 [file 44318_2024_155_MOESM7_ESM.zip › Fig 5/Fig 5E/5E P-p38.tif]

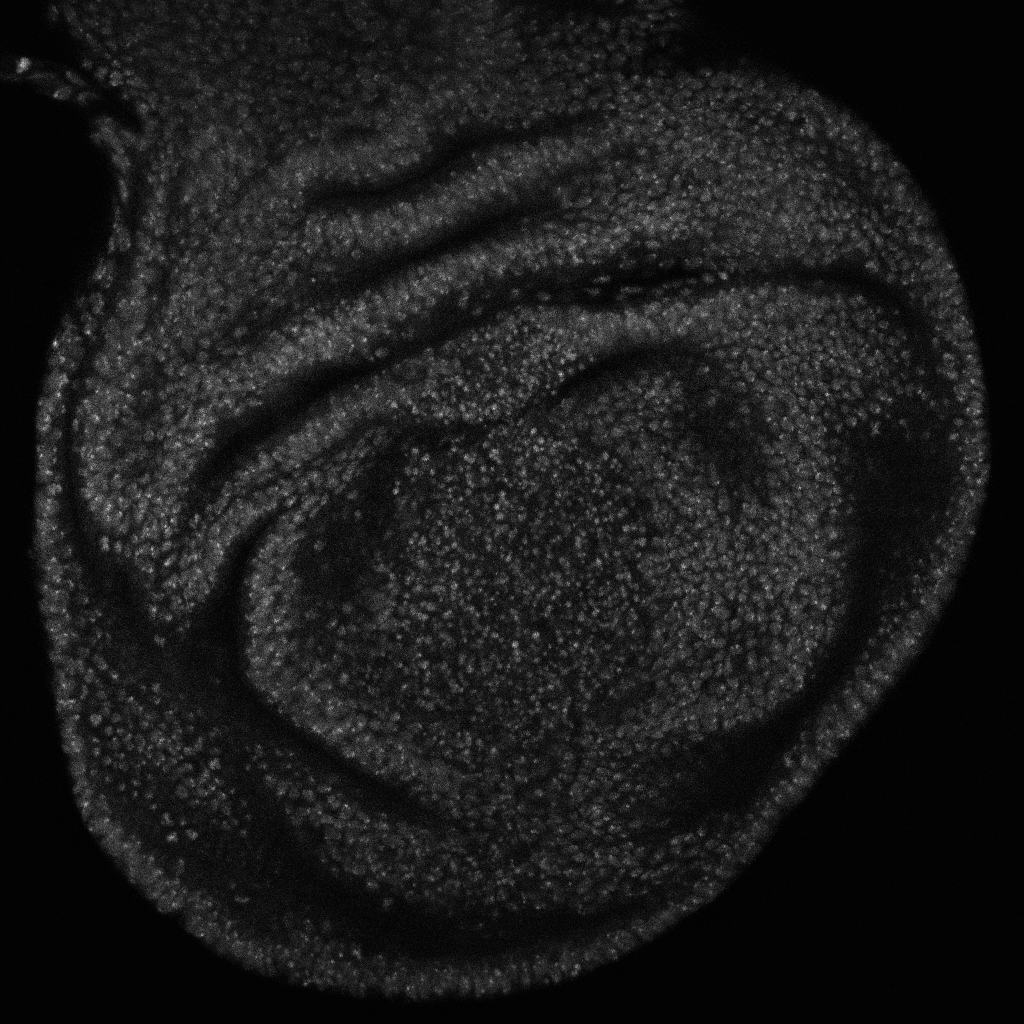

Supplement: Supplementary file 7 — Source data Fig. 5 [file 44318_2024_155_MOESM7_ESM.zip › Fig 5/Fig 5E/5E nuclei.tif]

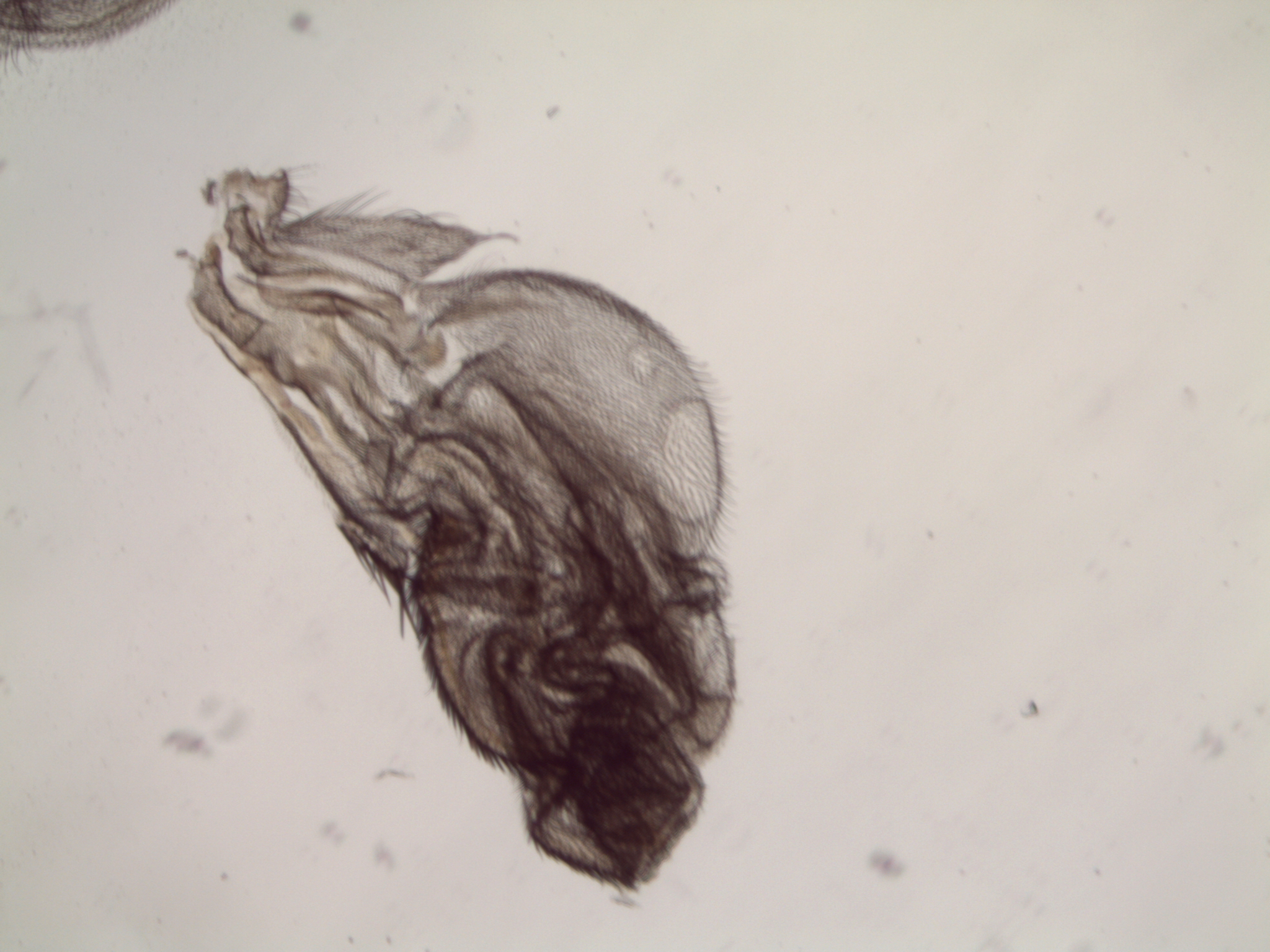

Supplement: Supplementary file 7 — Source data Fig. 5 [file 44318_2024_155_MOESM7_ESM.zip › Fig 5/Fig 5B/5B strong.jpg]

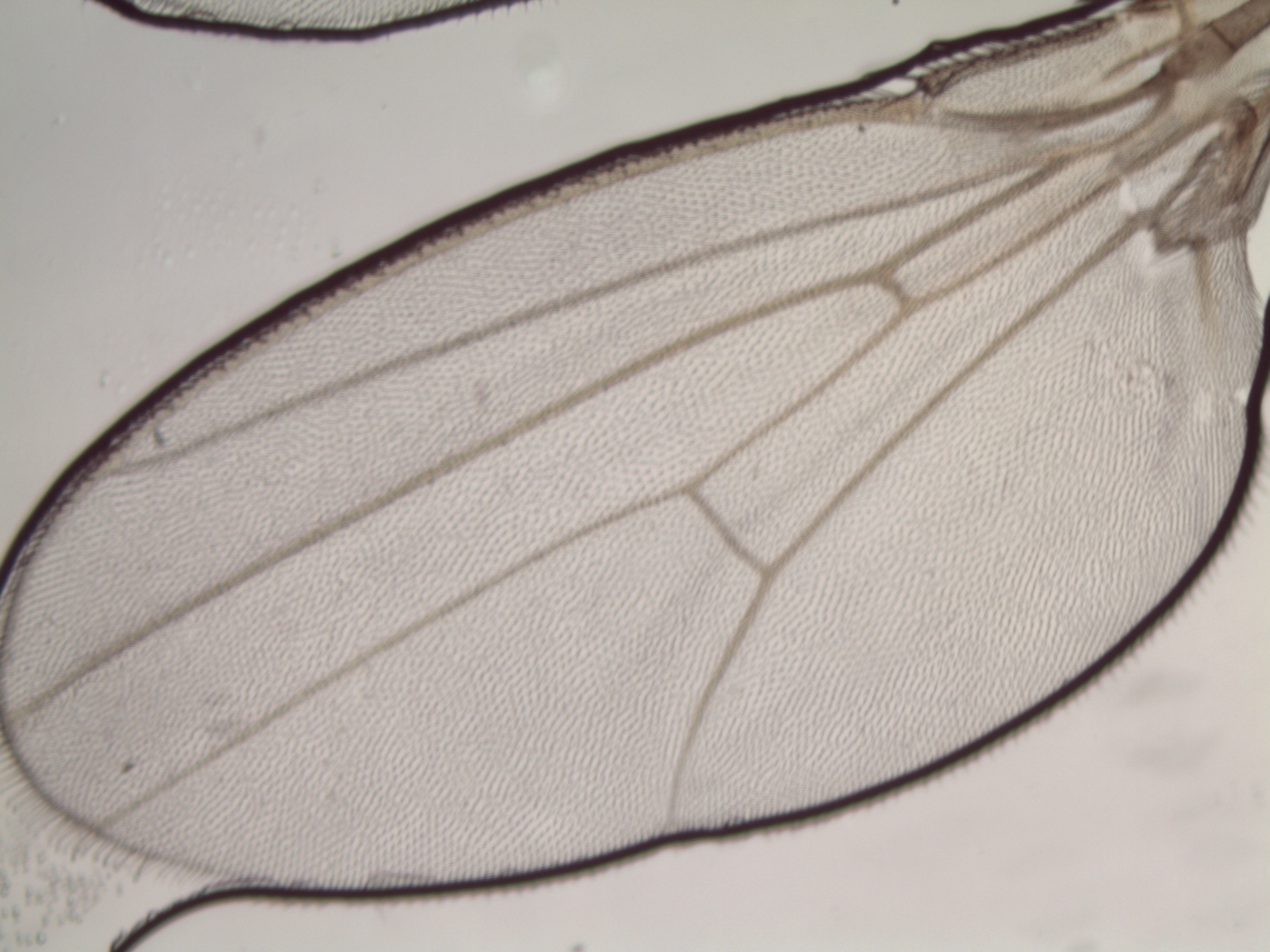

Supplement: Supplementary file 7 — Source data Fig. 5 [file 44318_2024_155_MOESM7_ESM.zip › Fig 5/Fig 5B/5B regenerated.tif]

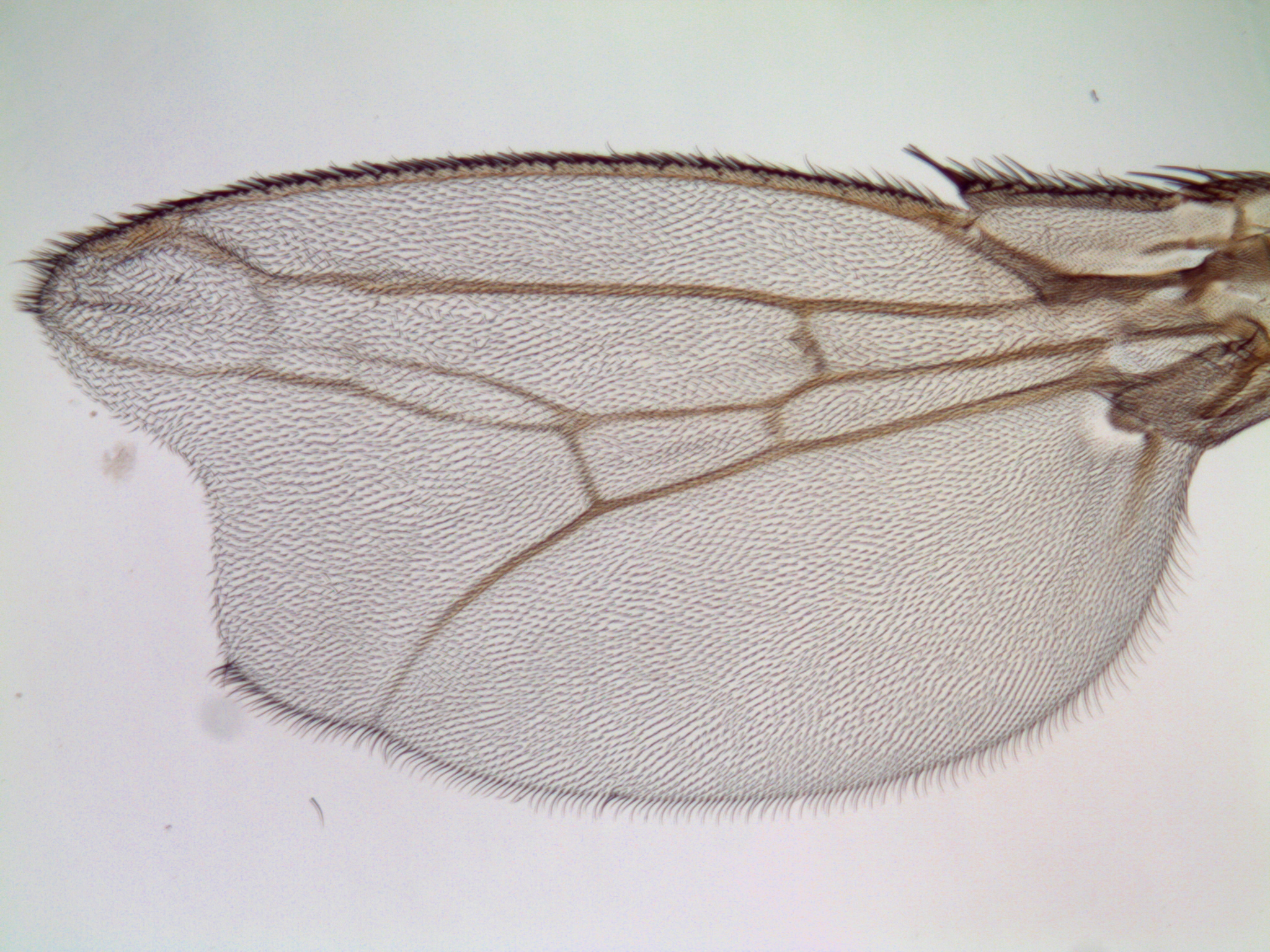

Supplement: Supplementary file 7 — Source data Fig. 5 [file 44318_2024_155_MOESM7_ESM.zip › Fig 5/Fig 5B/5B mild.tif]

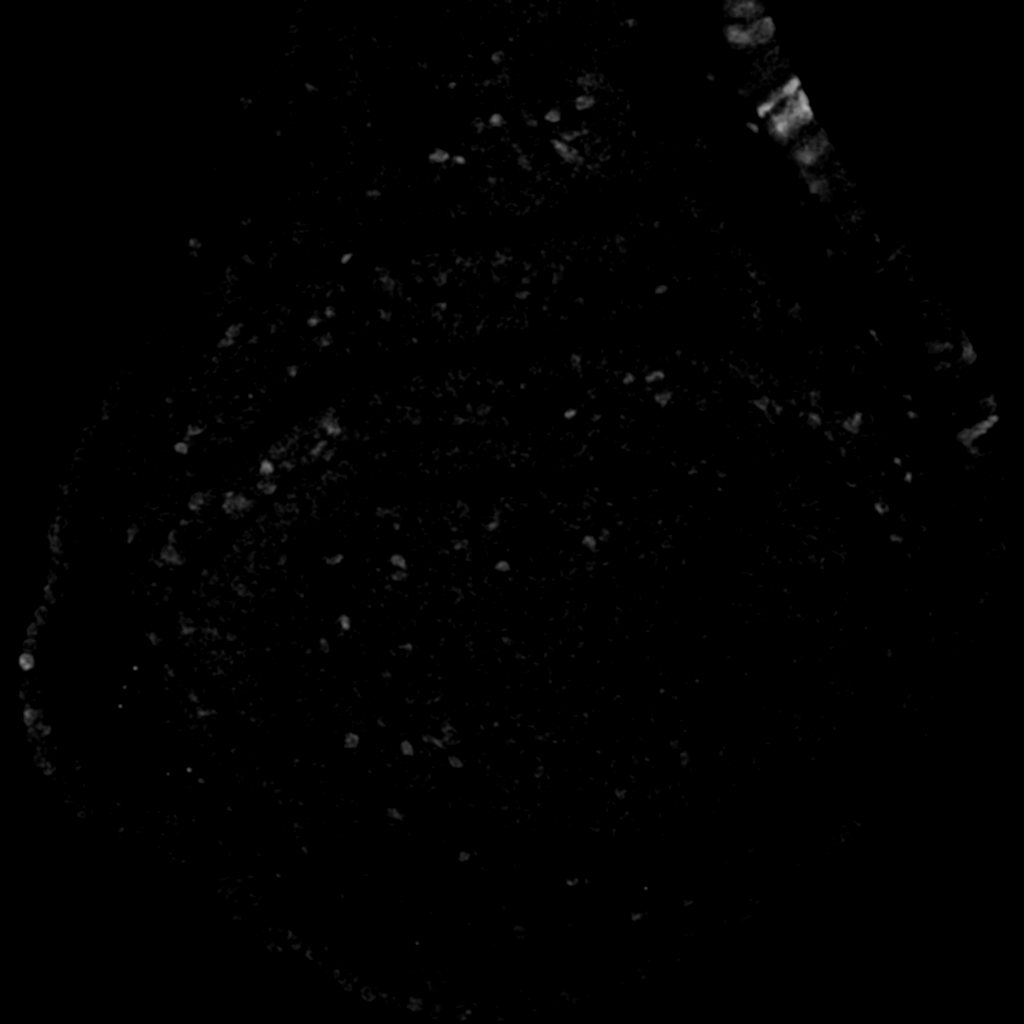

Supplement: Supplementary file 7 — Source data Fig. 5 [file 44318_2024_155_MOESM7_ESM.zip › Fig 5/Fig 5C/5C P-p38.tif]

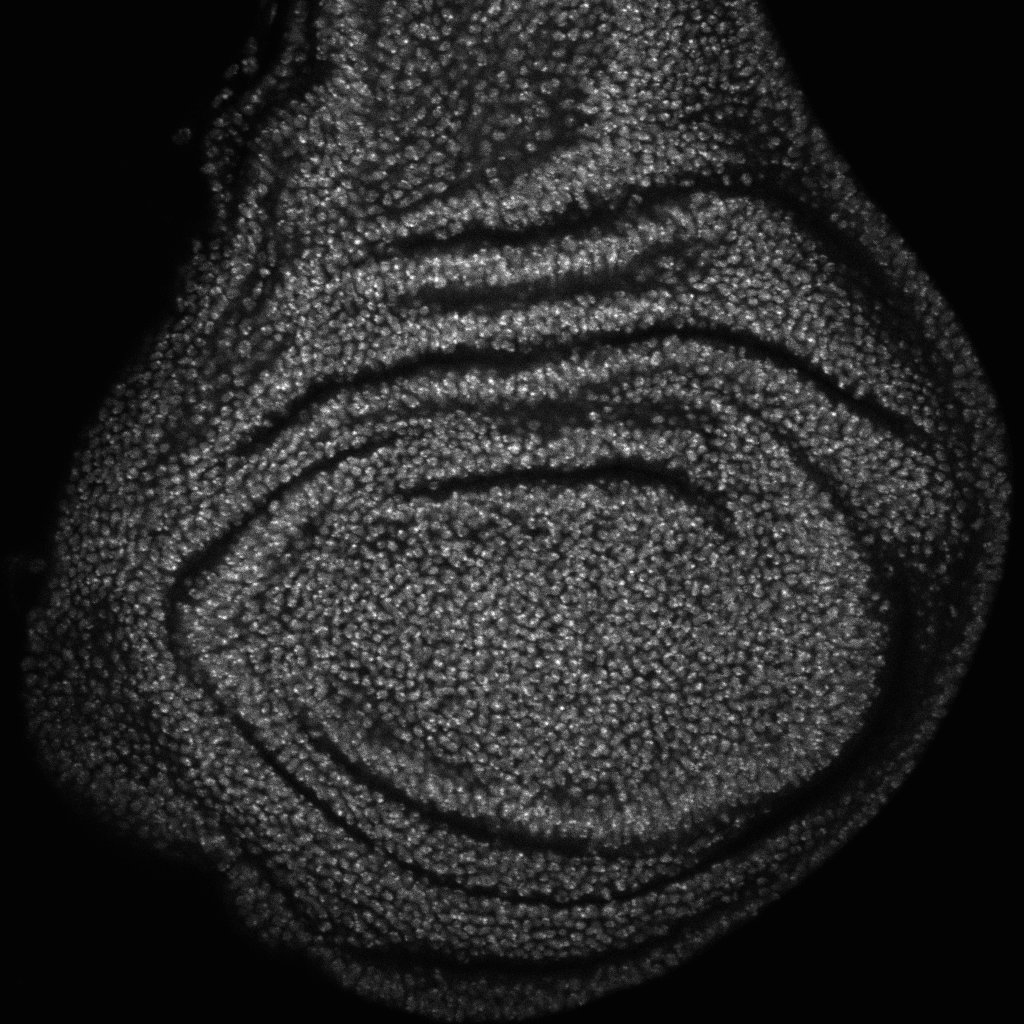

Supplement: Supplementary file 7 — Source data Fig. 5 [file 44318_2024_155_MOESM7_ESM.zip › Fig 5/Fig 5C/5C nuclei.tif]

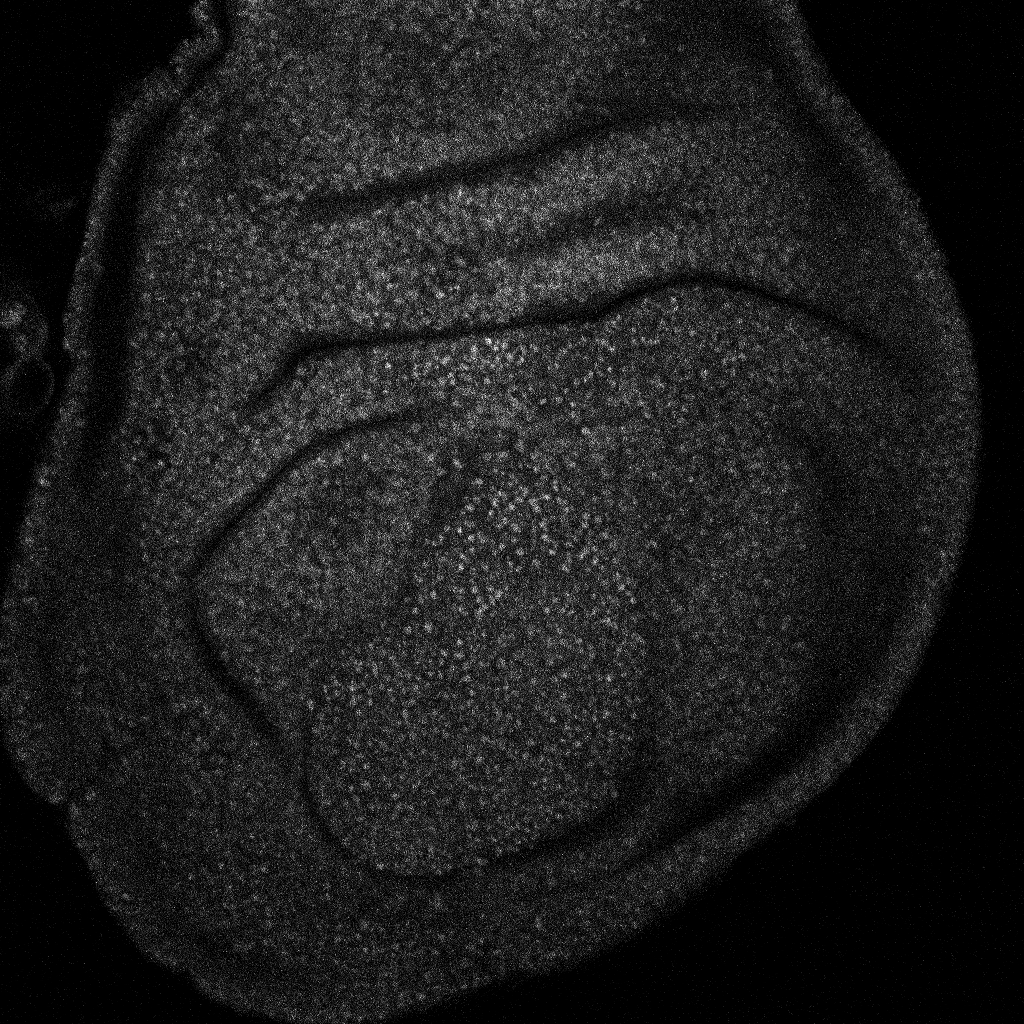

Supplement: Supplementary file 7 — Source data Fig. 5 [file 44318_2024_155_MOESM7_ESM.zip › Fig 5/Fig 5D/5D nuclei.tif]

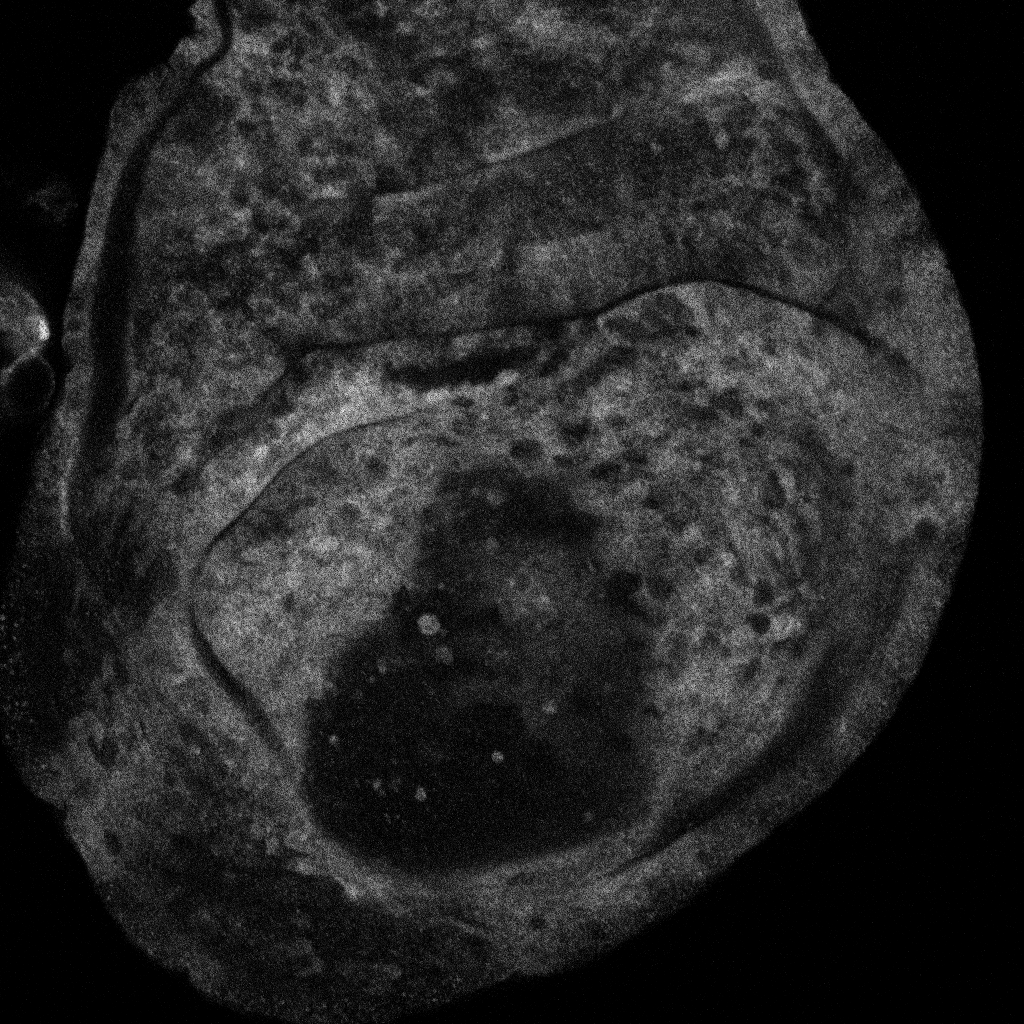

Supplement: Supplementary file 7 — Source data Fig. 5 [file 44318_2024_155_MOESM7_ESM.zip › Fig 5/Fig 5D/5D P-p38.tif]

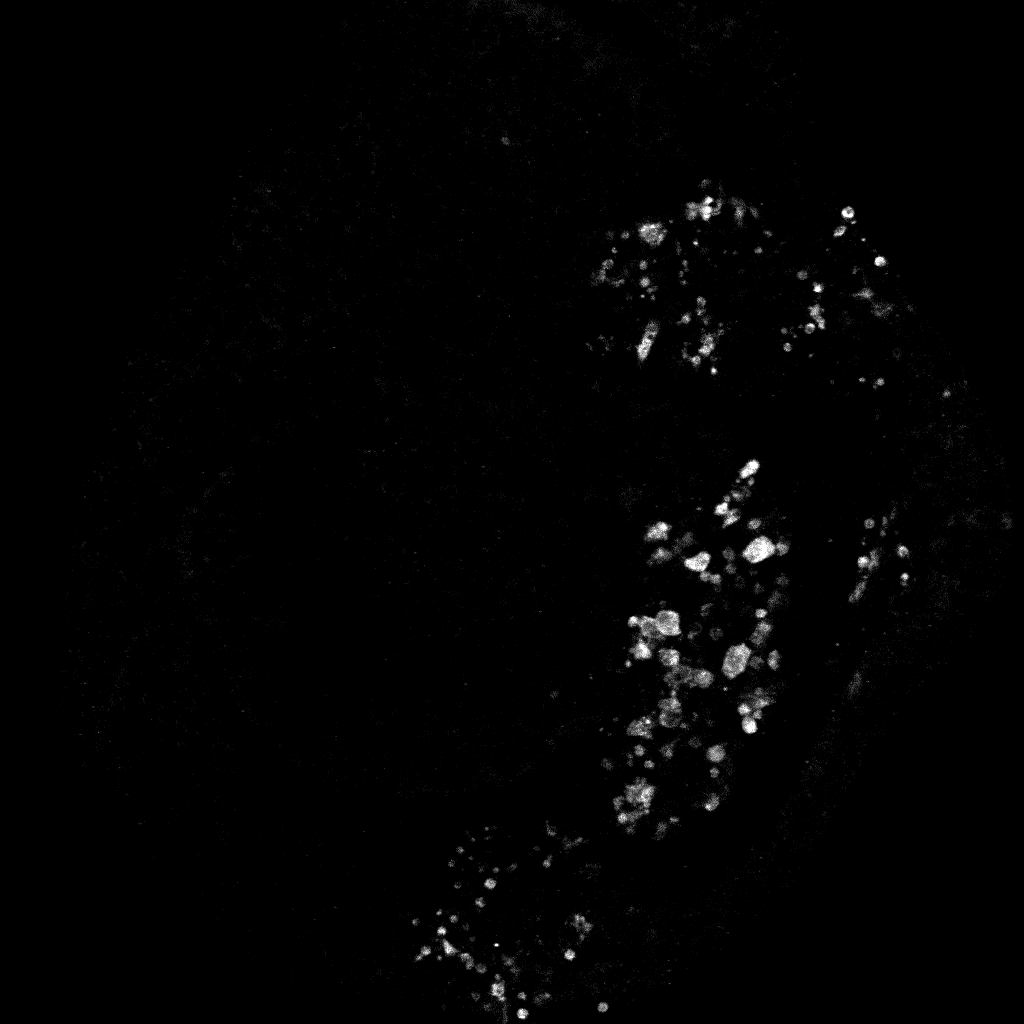

Supplement: Supplementary file 8 — EV Figures Source Data [file 44318_2024_155_MOESM8_ESM.zip › Fig. EV1/Fig EV1 A/dcp1 egr weak.tif]

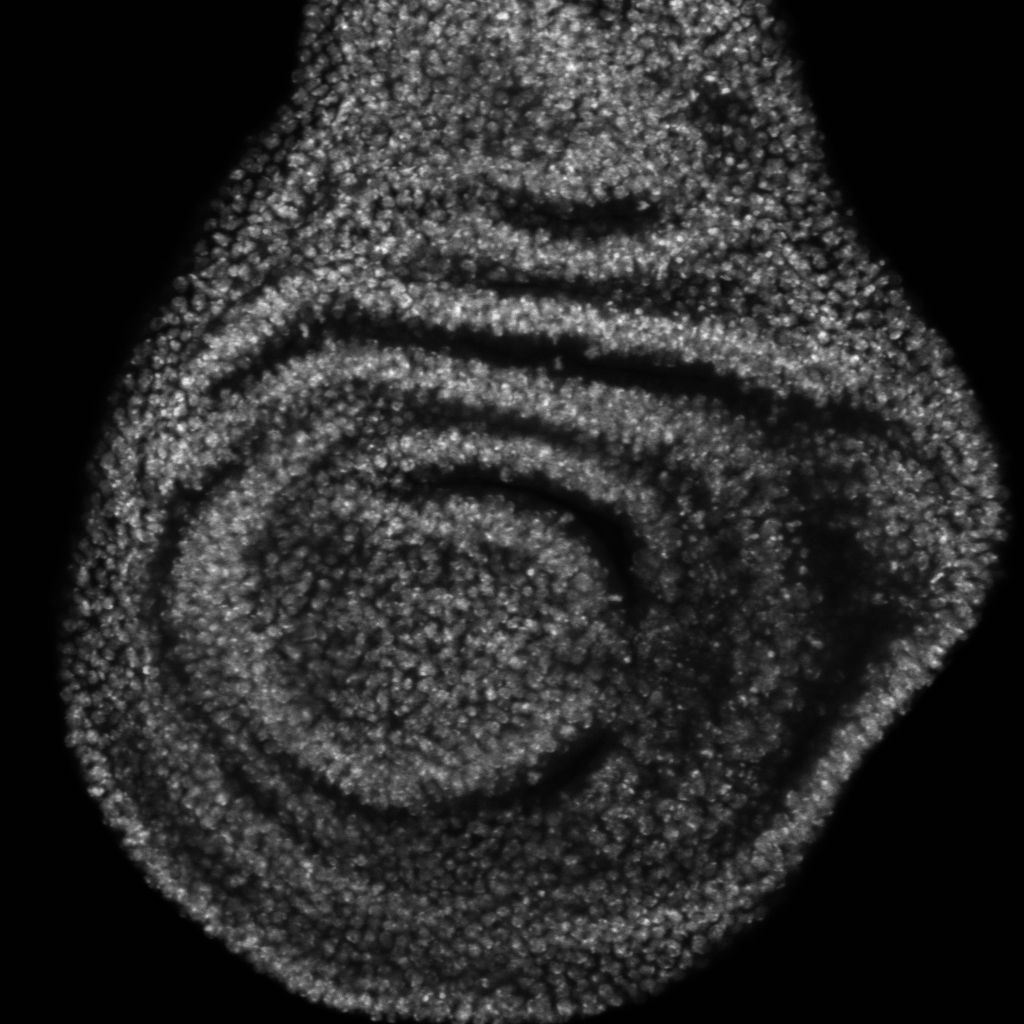

Supplement: Supplementary file 8 — EV Figures Source Data [file 44318_2024_155_MOESM8_ESM.zip › Fig. EV1/Fig EV1 A/nuclei egr weak.tif]

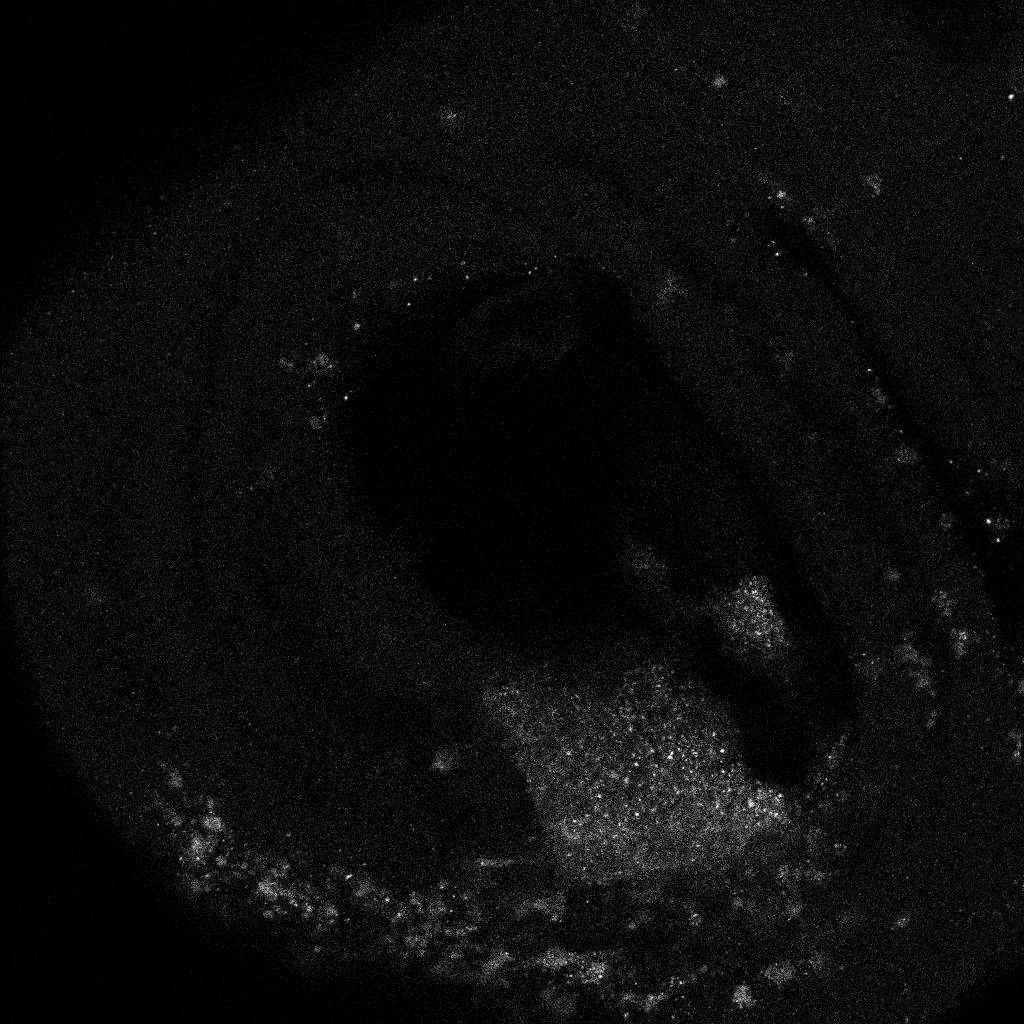

Supplement: Supplementary file 8 — EV Figures Source Data [file 44318_2024_155_MOESM8_ESM.zip › Fig. EV1/Fig EV1 B/UAS-egrw UAS-RNAiwgn hh-Gal4 mmp1-488 dcp1-532 topro-farred.lif - Series005.tif]

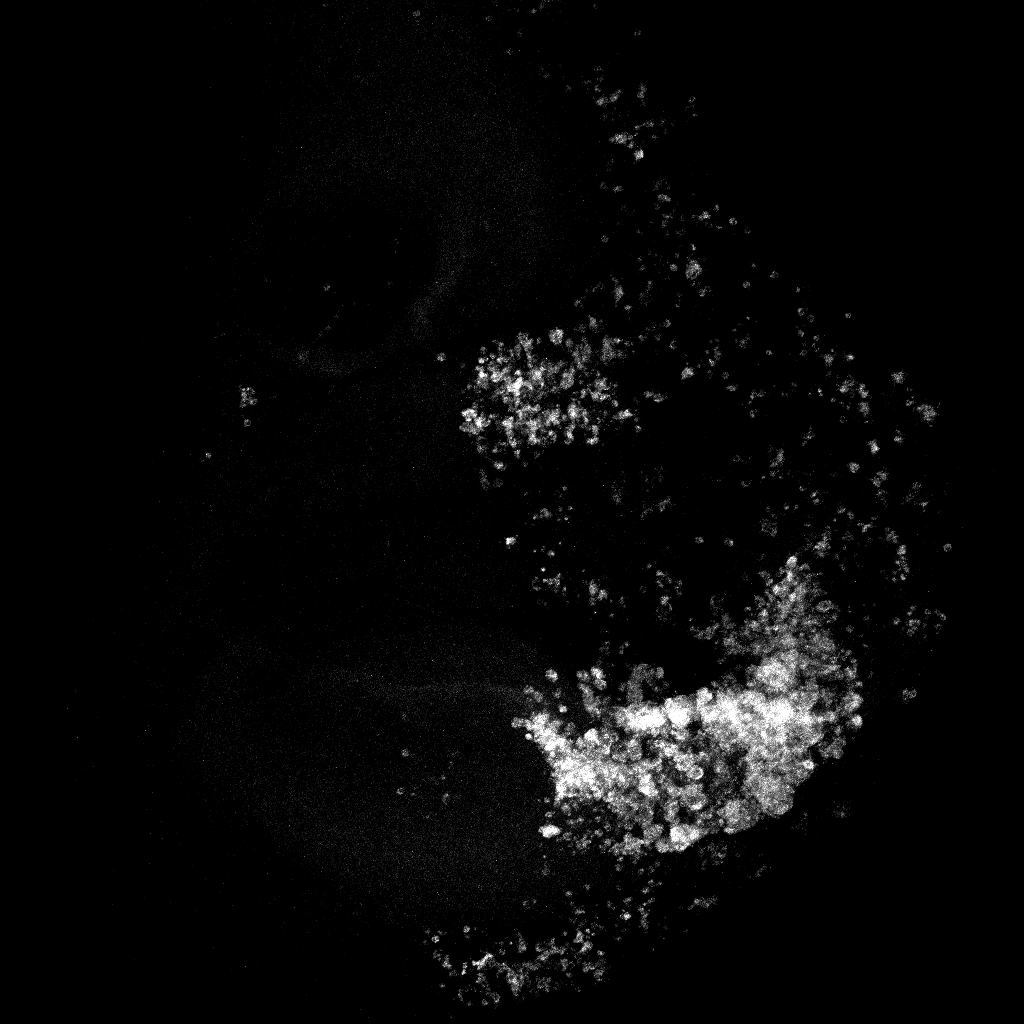

Supplement: Supplementary file 8 — EV Figures Source Data [file 44318_2024_155_MOESM8_ESM.zip › Fig. EV1/Fig EV1 C/dcp1 wng rnai bloom.tif]

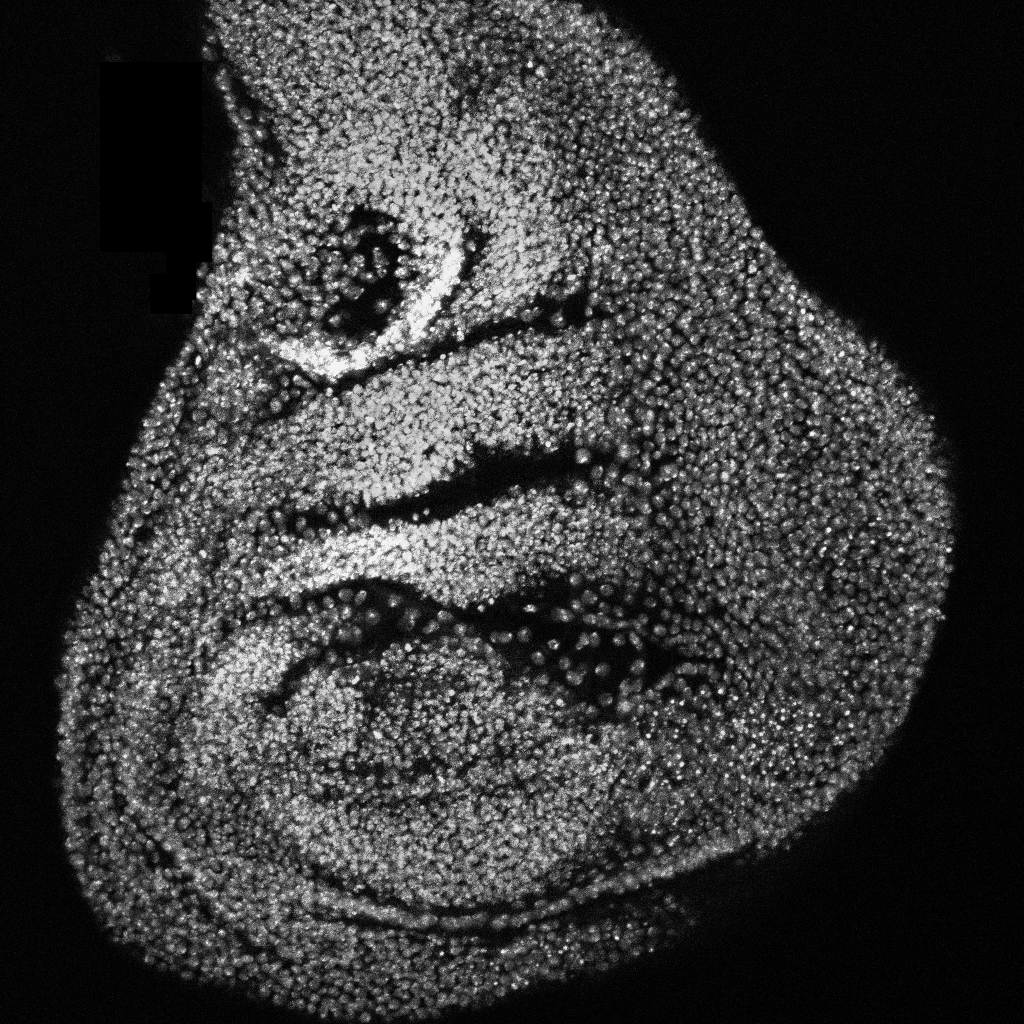

Supplement: Supplementary file 8 — EV Figures Source Data [file 44318_2024_155_MOESM8_ESM.zip › Fig. EV1/Fig EV1 C/nuclei wng bloom.tif]

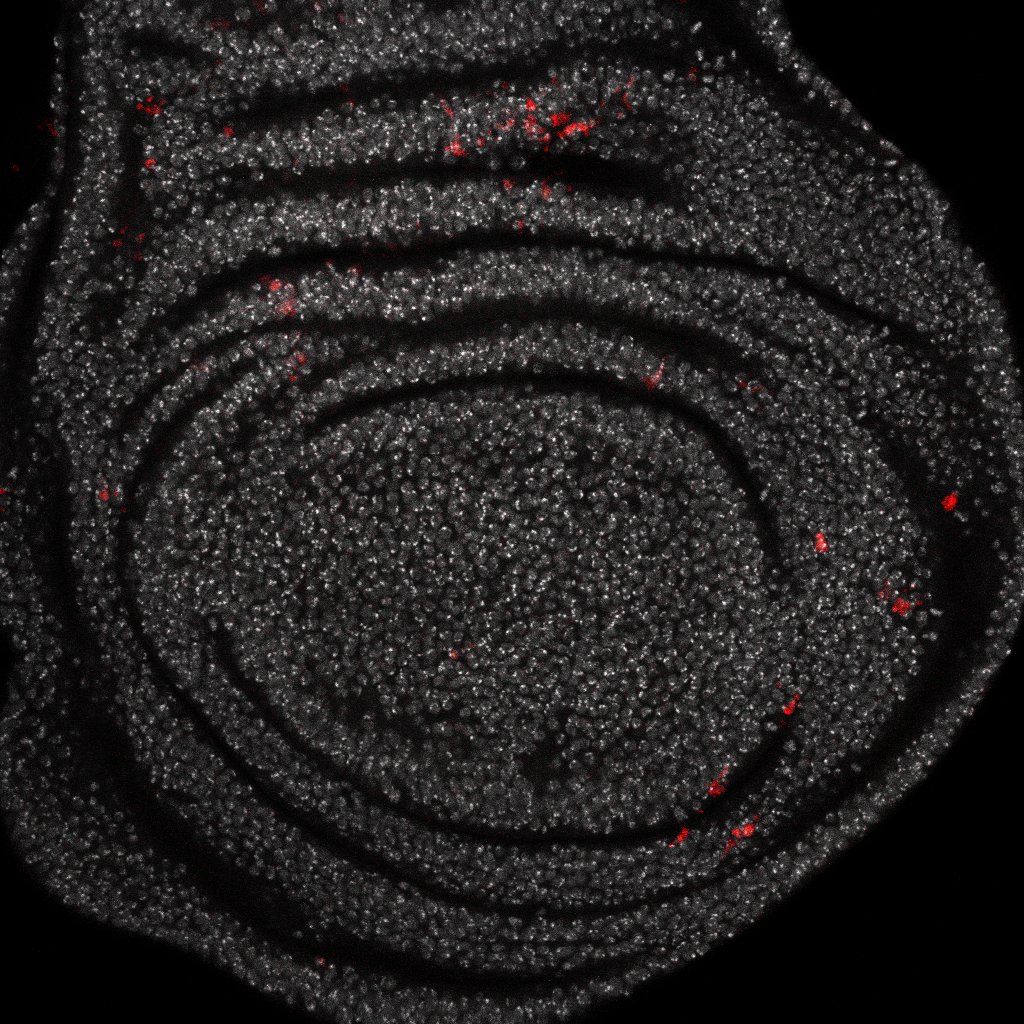

Supplement: Supplementary file 8 — EV Figures Source Data [file 44318_2024_155_MOESM8_ESM.zip › Fig. EV1/Fig EV1 D/composite rnai vienn alone.jpg]

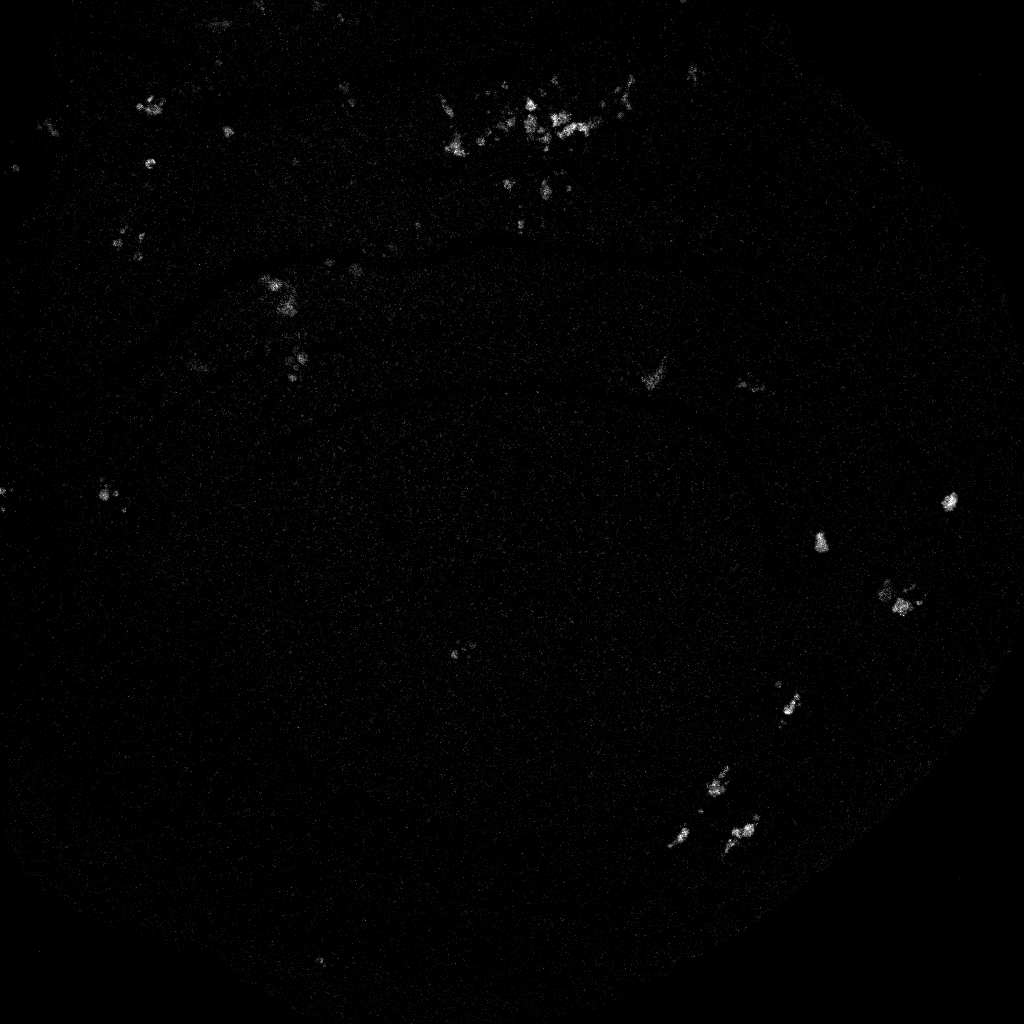

Supplement: Supplementary file 8 — EV Figures Source Data [file 44318_2024_155_MOESM8_ESM.zip › Fig. EV1/Fig EV1 D/dcp1 rnai viena alone.tif]

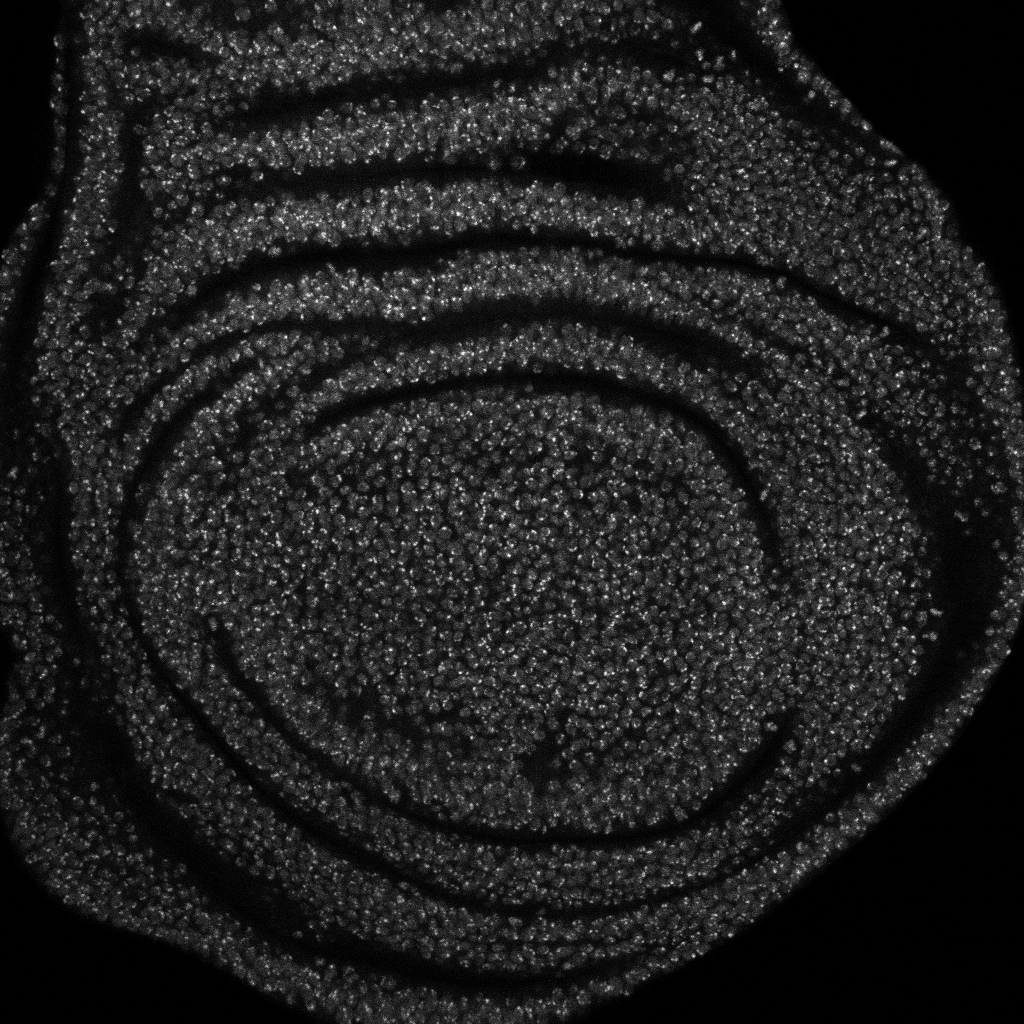

Supplement: Supplementary file 8 — EV Figures Source Data [file 44318_2024_155_MOESM8_ESM.zip › Fig. EV1/Fig EV1 D/nuclei rnai vienna alone.tif]

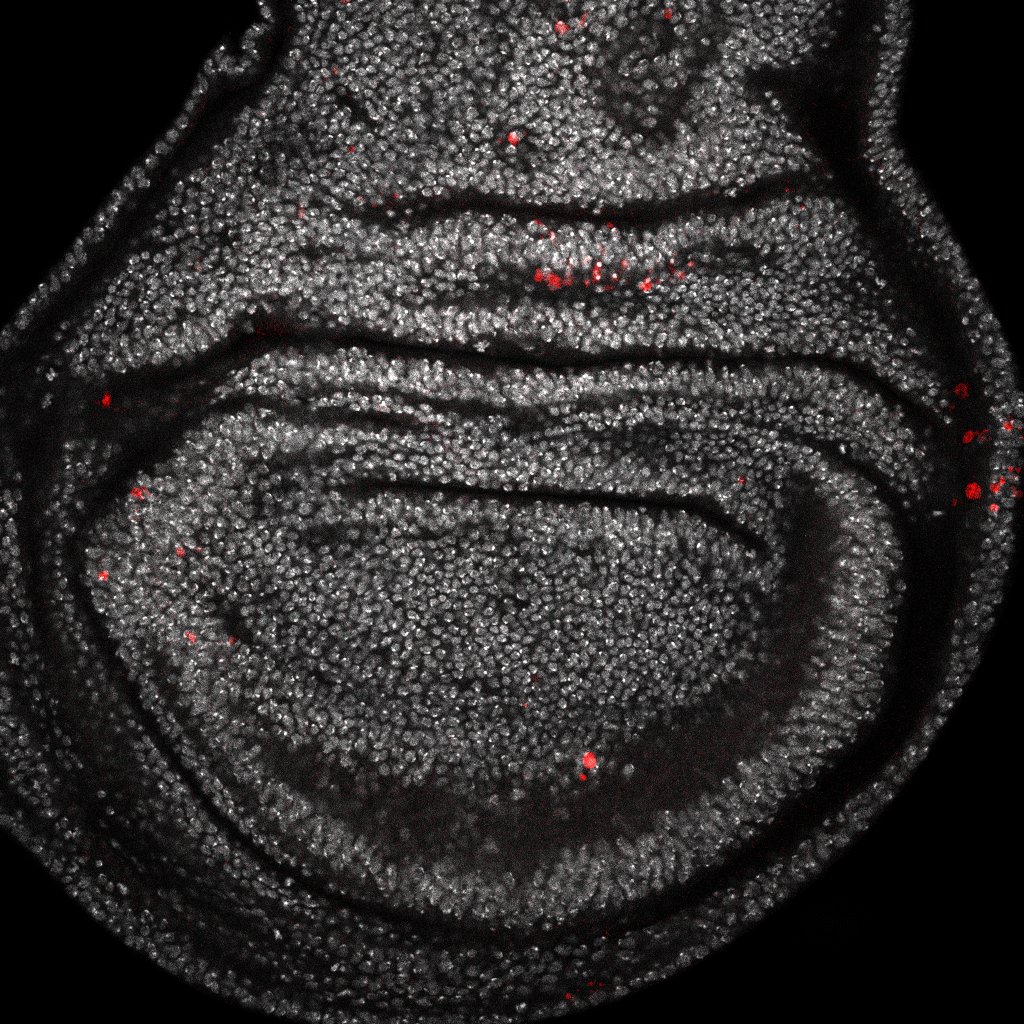

Supplement: Supplementary file 8 — EV Figures Source Data [file 44318_2024_155_MOESM8_ESM.zip › Fig. EV1/Fig EV1 E/Composite RNAI WGN BLOOM ALONE.jpg]

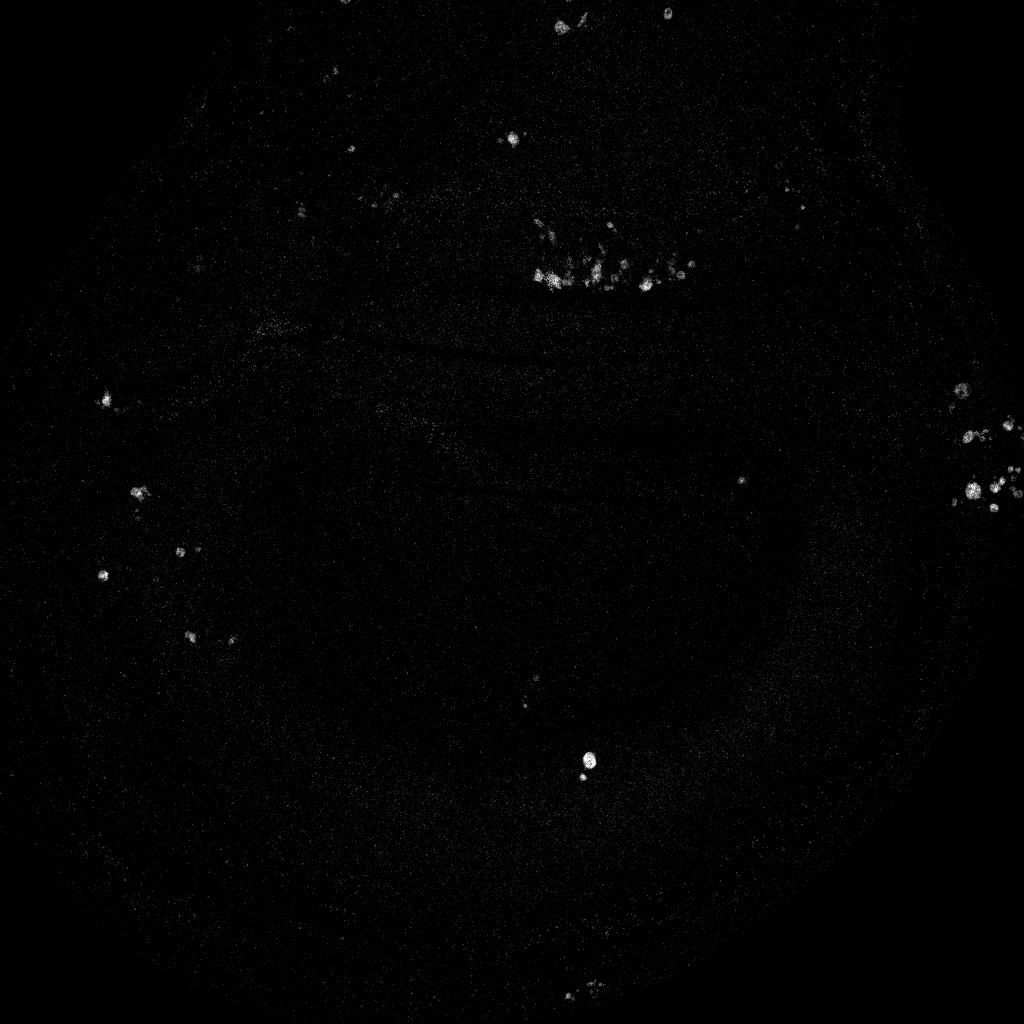

Supplement: Supplementary file 8 — EV Figures Source Data [file 44318_2024_155_MOESM8_ESM.zip › Fig. EV1/Fig EV1 E/DCP1 RNAI WGN BLOOM ALONE.tif]

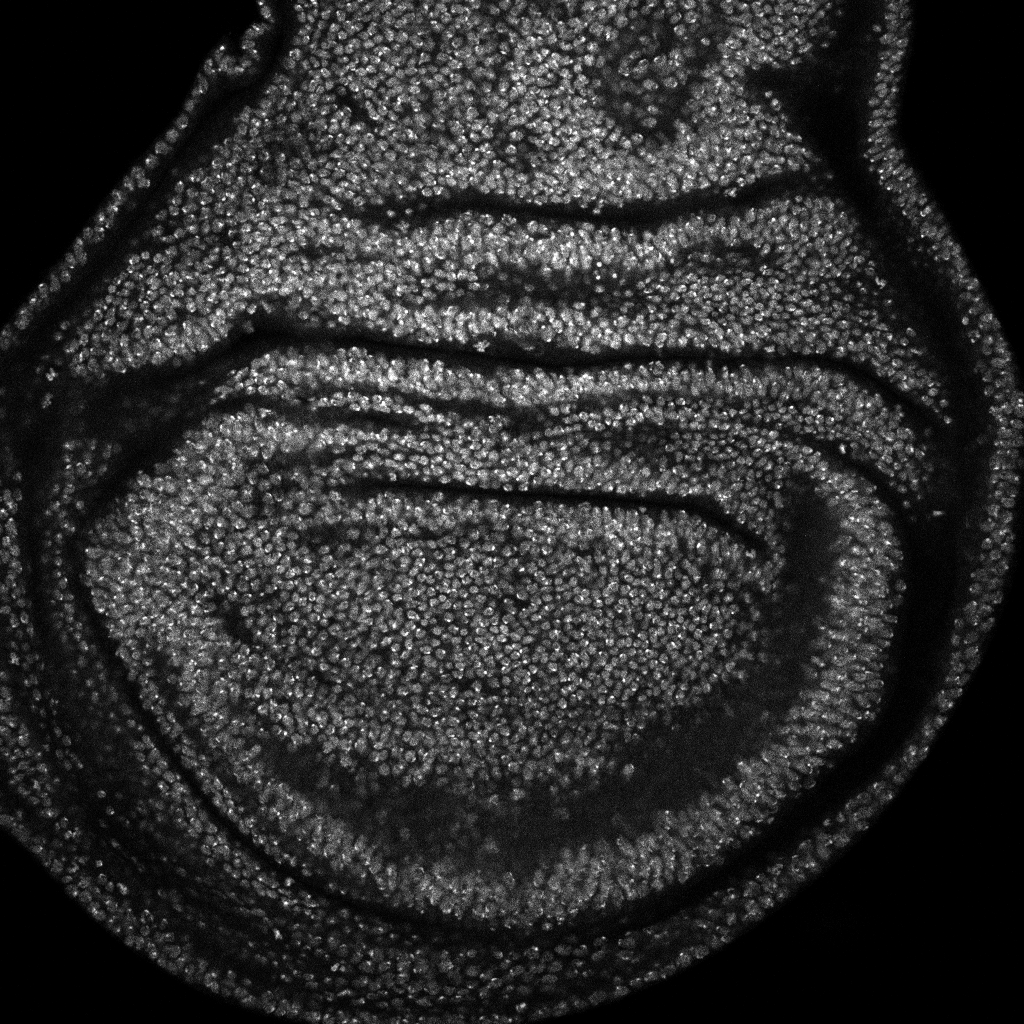

Supplement: Supplementary file 8 — EV Figures Source Data [file 44318_2024_155_MOESM8_ESM.zip › Fig. EV1/Fig EV1 E/NUCLEI Rnai wgn bloom alone.tif]

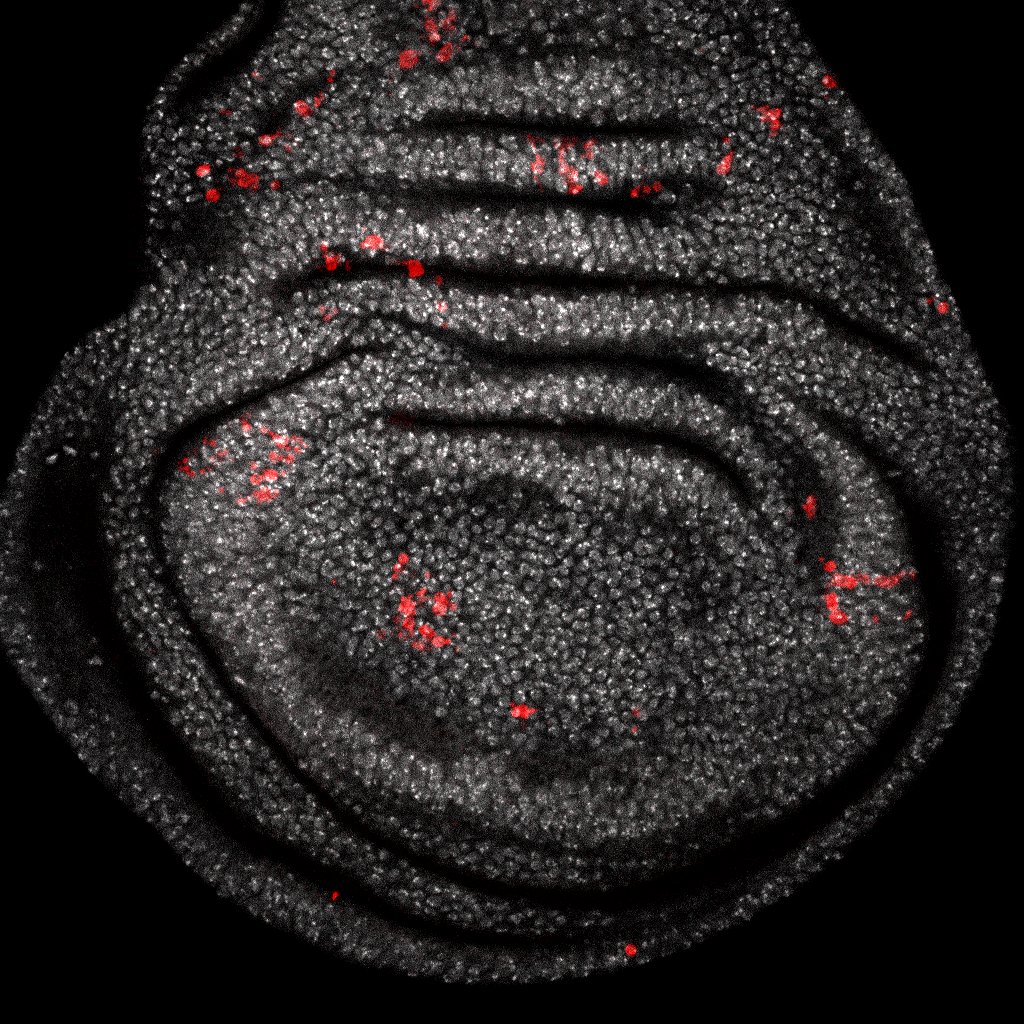

Supplement: Supplementary file 8 — EV Figures Source Data [file 44318_2024_155_MOESM8_ESM.zip › Fig. EV1/Fig EV1 F/Composite egr weak rnai grnd new.jpg]

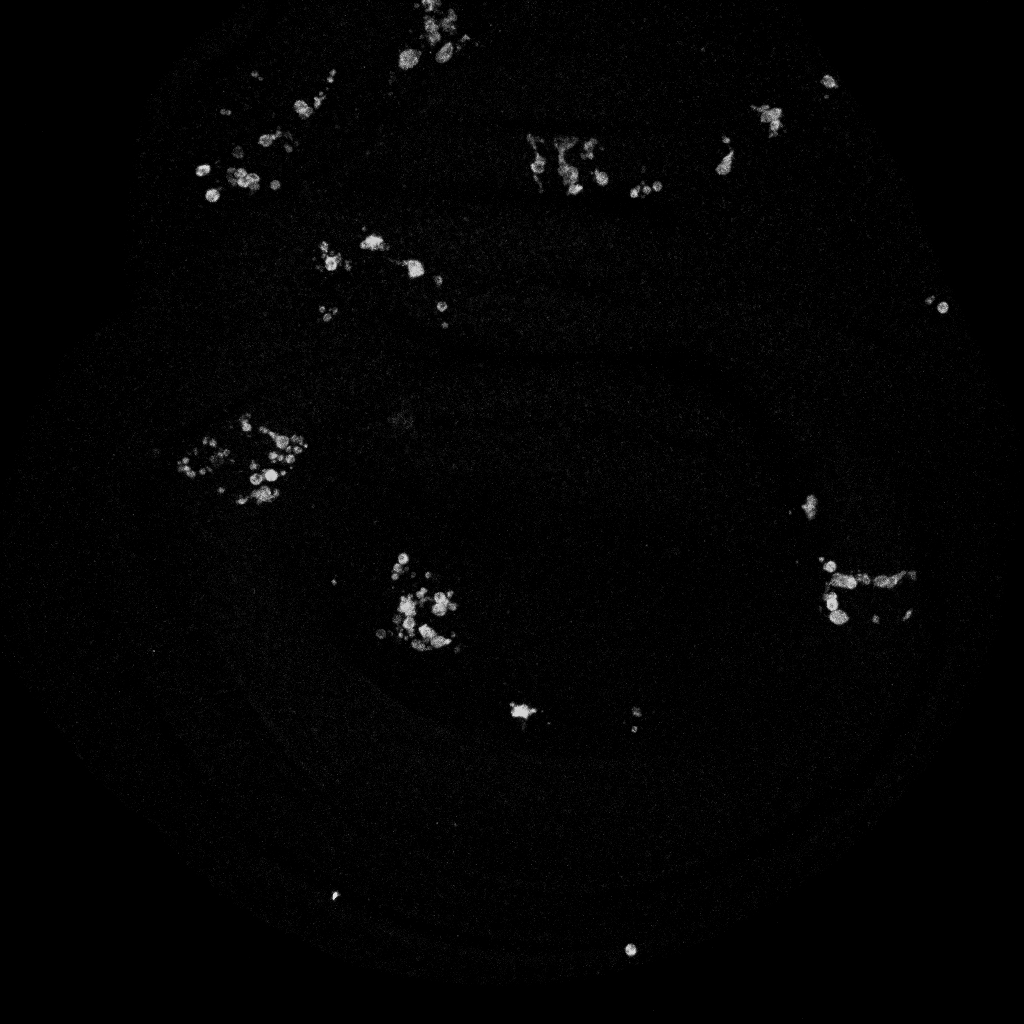

Supplement: Supplementary file 8 — EV Figures Source Data [file 44318_2024_155_MOESM8_ESM.zip › Fig. EV1/Fig EV1 F/hh egr weak rnai grnd new.tif]

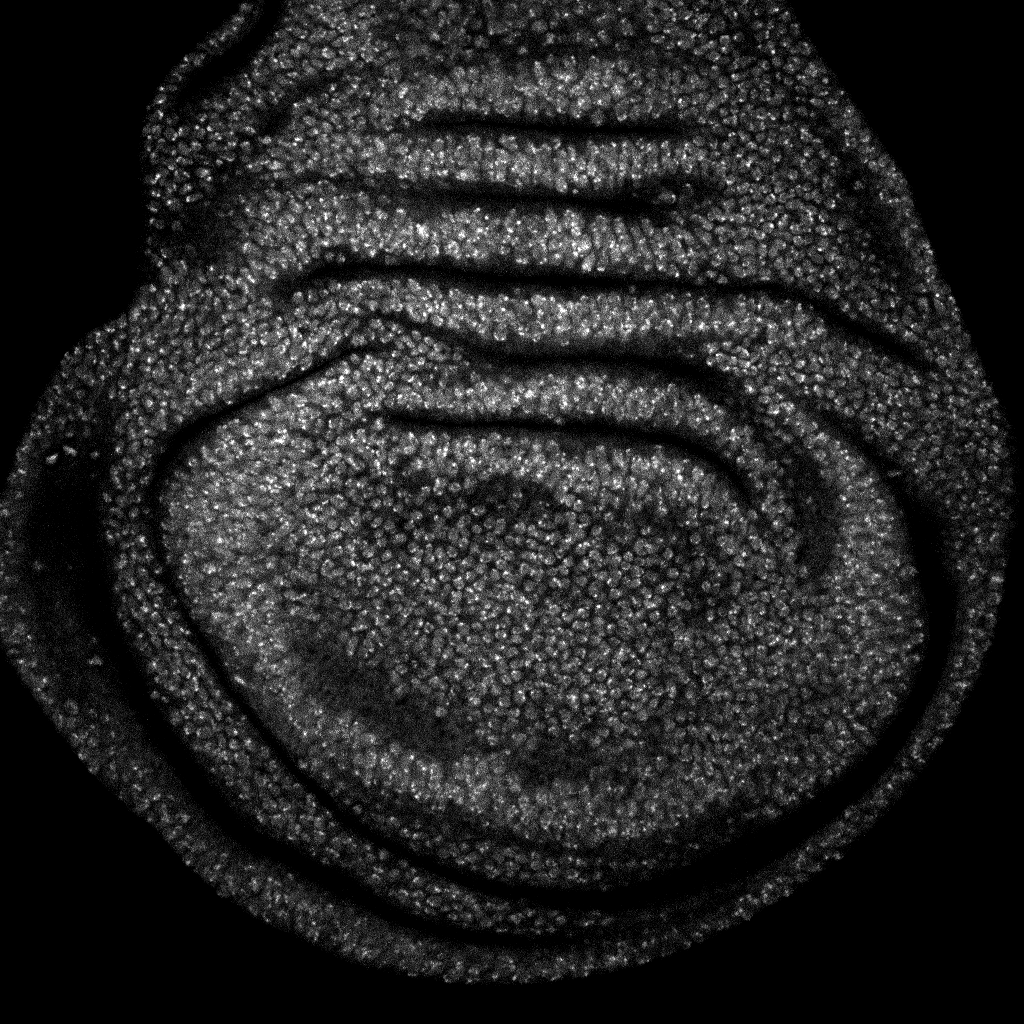

Supplement: Supplementary file 8 — EV Figures Source Data [file 44318_2024_155_MOESM8_ESM.zip › Fig. EV1/Fig EV1 F/nuclei hh egr weak rnai grnd new.tif]

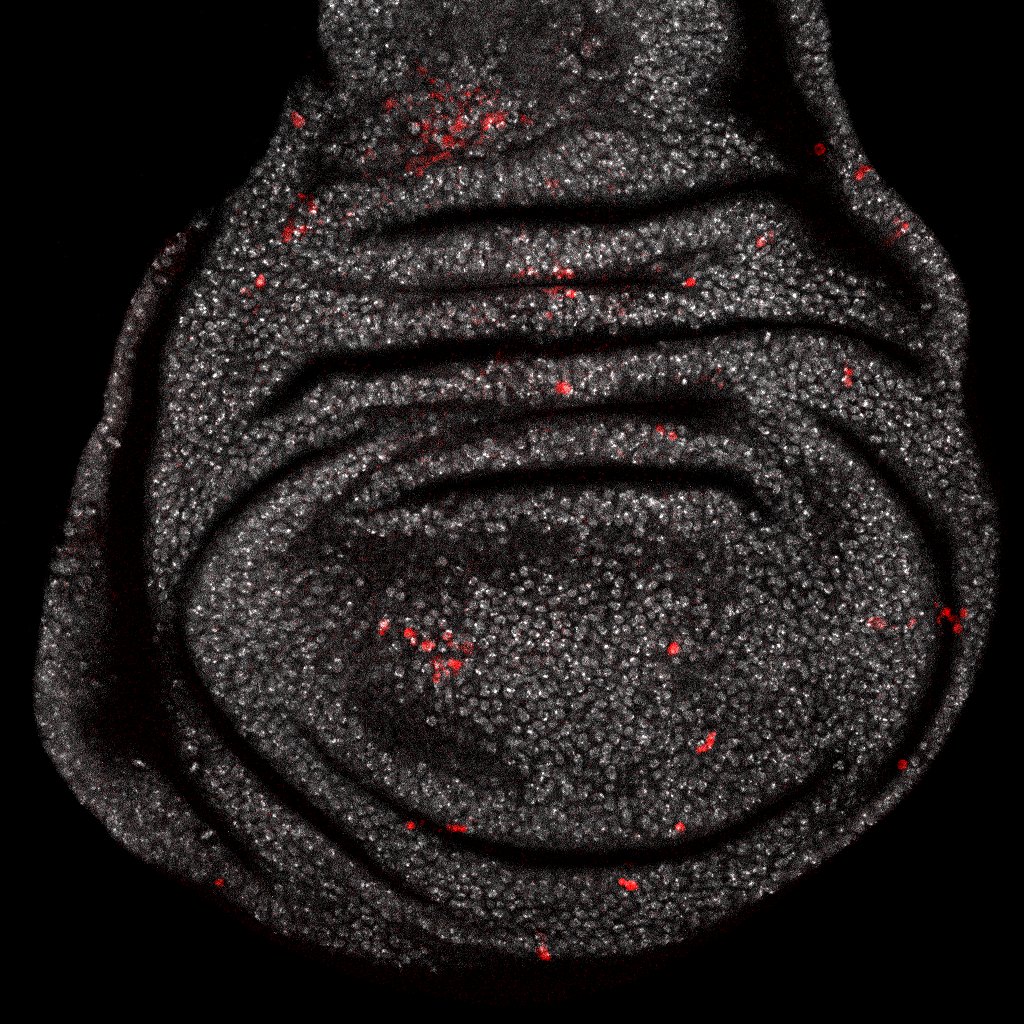

Supplement: Supplementary file 8 — EV Figures Source Data [file 44318_2024_155_MOESM8_ESM.zip › Fig. EV1/Fig EV1 G/Composite hh egr weak rnai traf2 new.jpg]

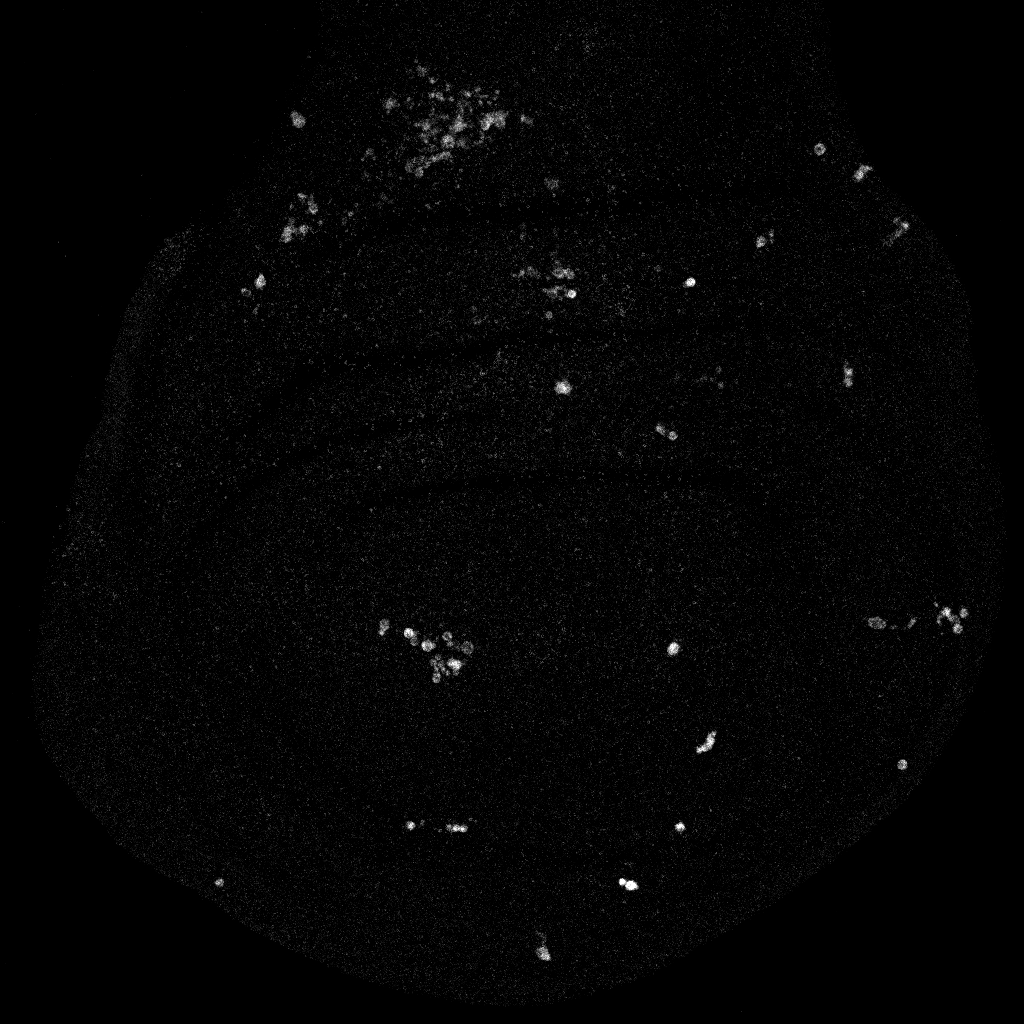

Supplement: Supplementary file 8 — EV Figures Source Data [file 44318_2024_155_MOESM8_ESM.zip › Fig. EV1/Fig EV1 G/dcp1 hh egrweak rrani traf2 new.tif]

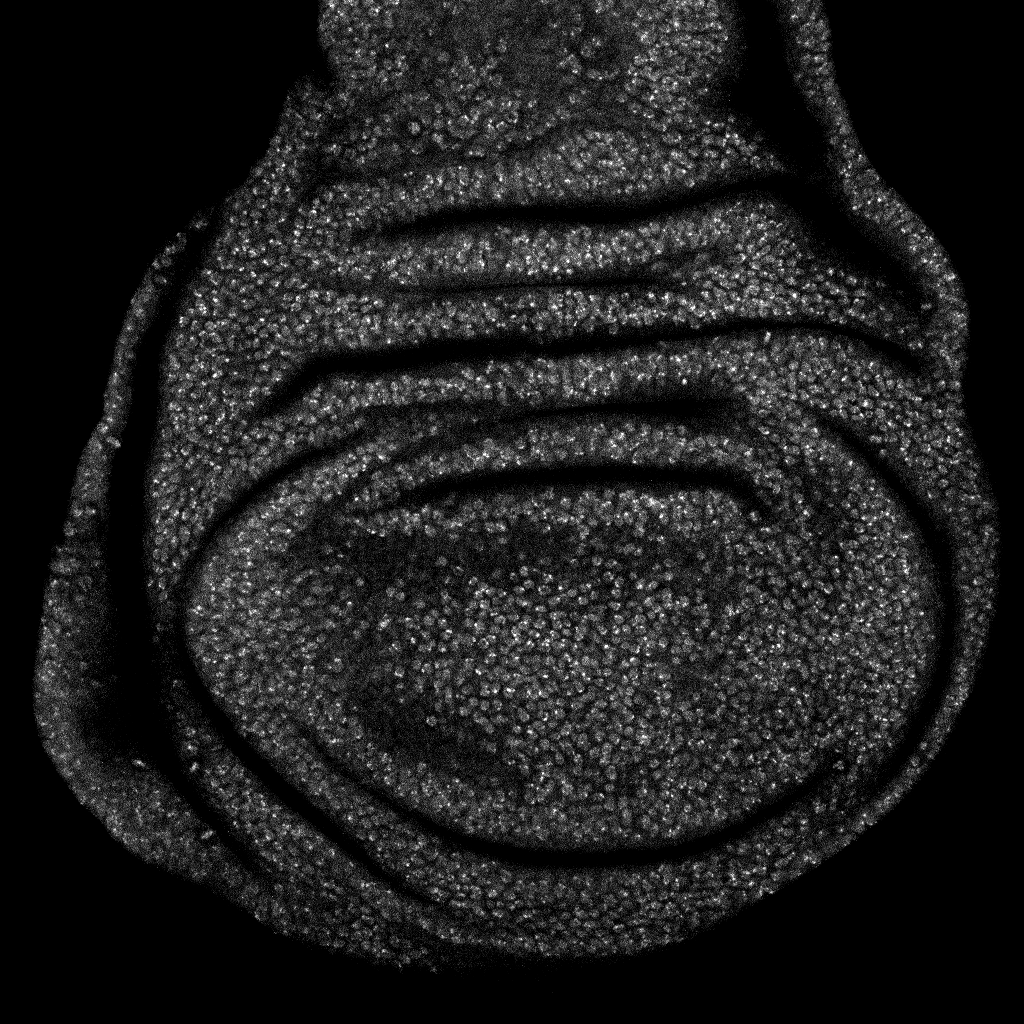

Supplement: Supplementary file 8 — EV Figures Source Data [file 44318_2024_155_MOESM8_ESM.zip › Fig. EV1/Fig EV1 G/nuclei hhegr weak rnai traf2 new.tif]

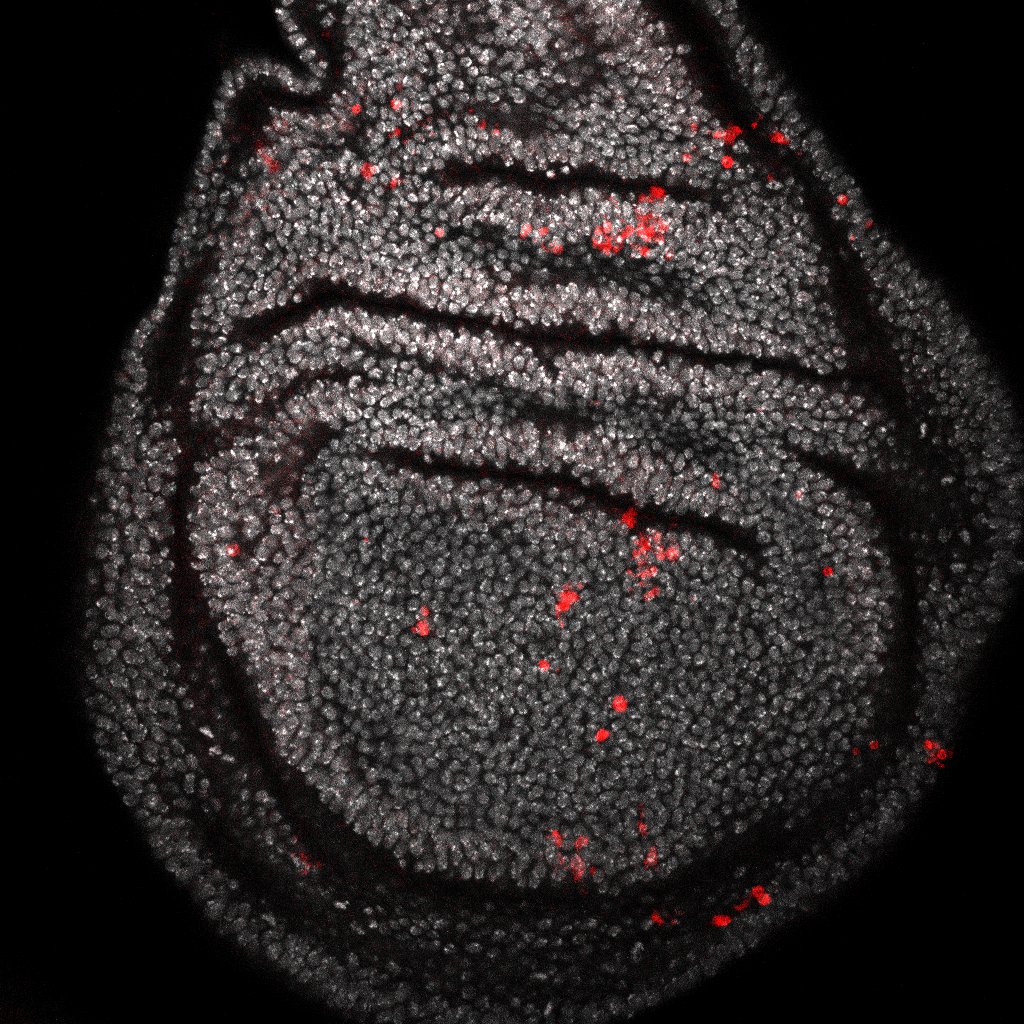

Supplement: Supplementary file 8 — EV Figures Source Data [file 44318_2024_155_MOESM8_ESM.zip › Fig. EV1/Fig EV1 H/Composite HH EGR WEAK RNAI TAK1 NEW.jpg]

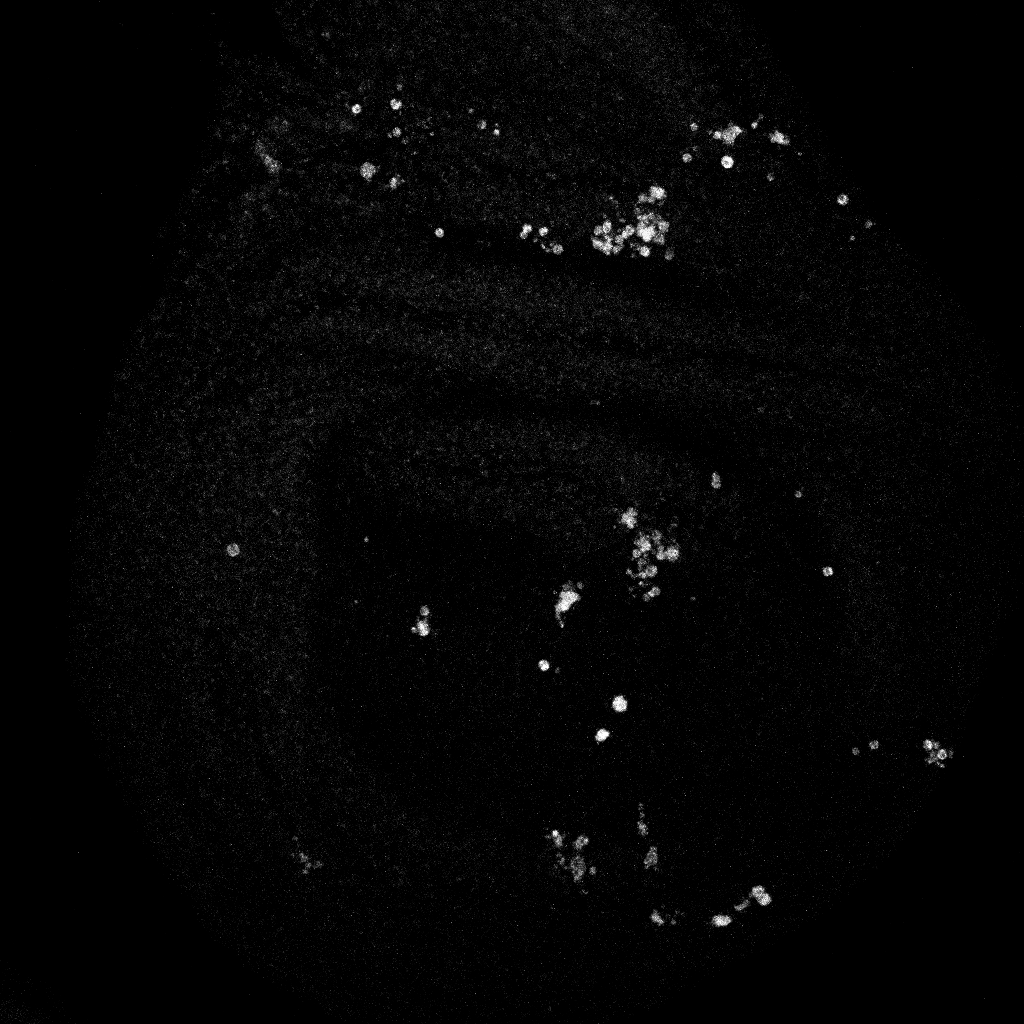

Supplement: Supplementary file 8 — EV Figures Source Data [file 44318_2024_155_MOESM8_ESM.zip › Fig. EV1/Fig EV1 H/DCP1 HH EGR WEAK RNAI TAK1 NEW.tif]

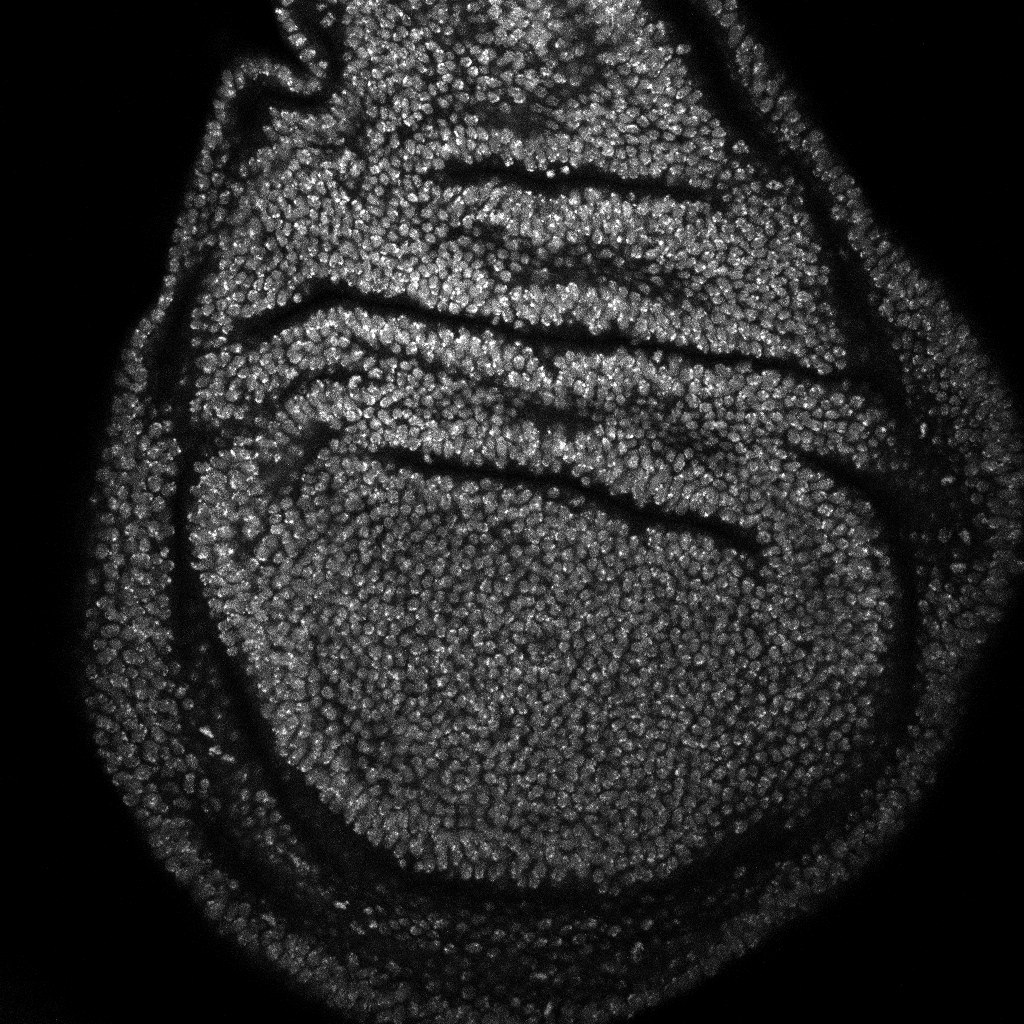

Supplement: Supplementary file 8 — EV Figures Source Data [file 44318_2024_155_MOESM8_ESM.zip › Fig. EV1/Fig EV1 H/NUCLEI HH EGR WEAK RNAI TAK1 NEW.tif]

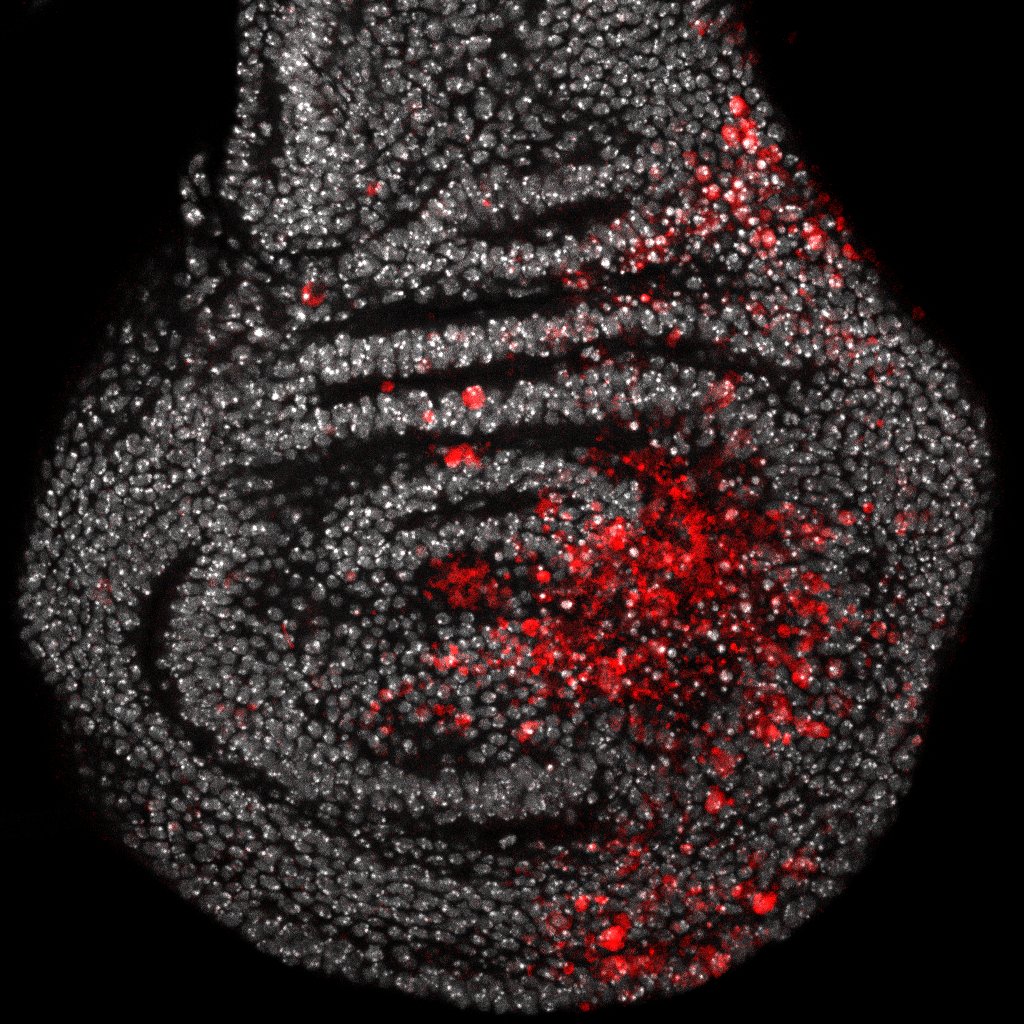

Supplement: Supplementary file 8 — EV Figures Source Data [file 44318_2024_155_MOESM8_ESM.zip › Fig. EV1/Fig EV1 I/Composite egr weak rnai traf1 new.jpg]

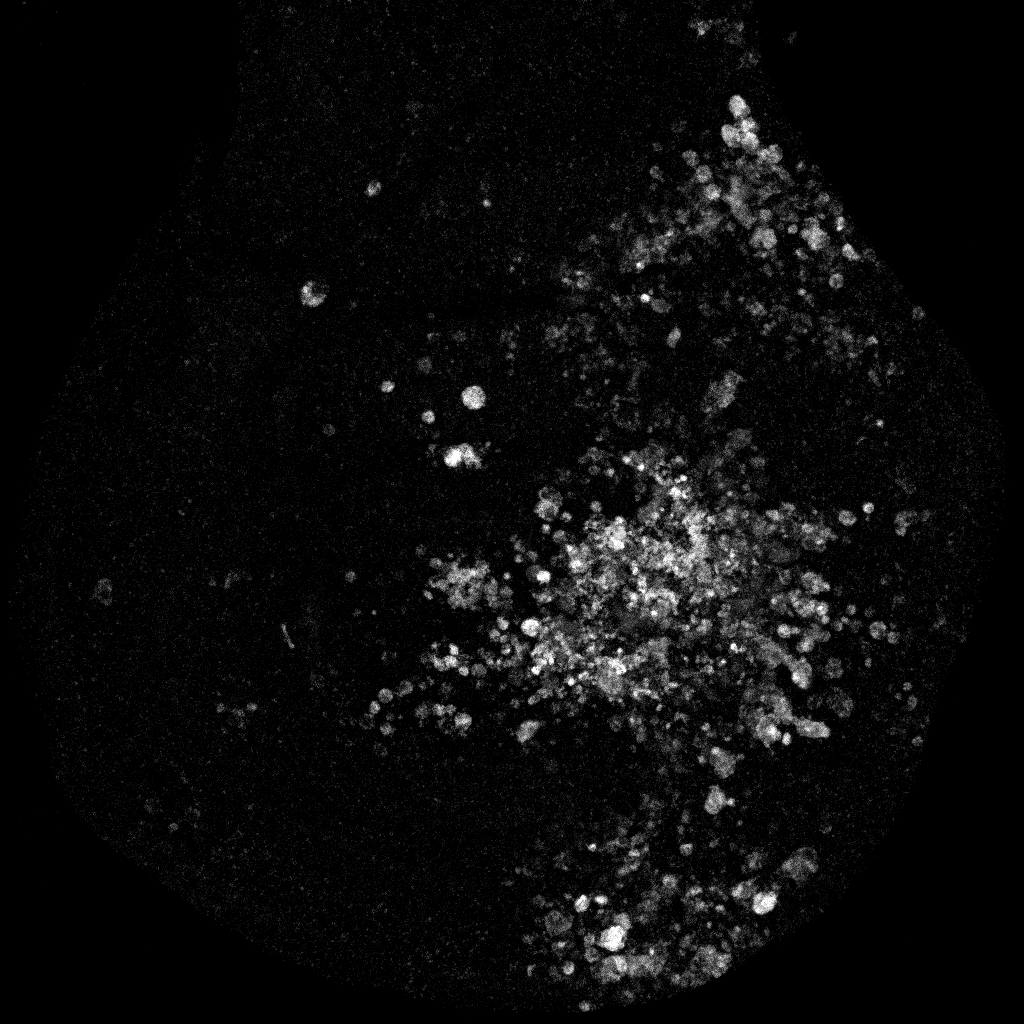

Supplement: Supplementary file 8 — EV Figures Source Data [file 44318_2024_155_MOESM8_ESM.zip › Fig. EV1/Fig EV1 I/dcp1 egr weak rnai traf 1 new.tif]

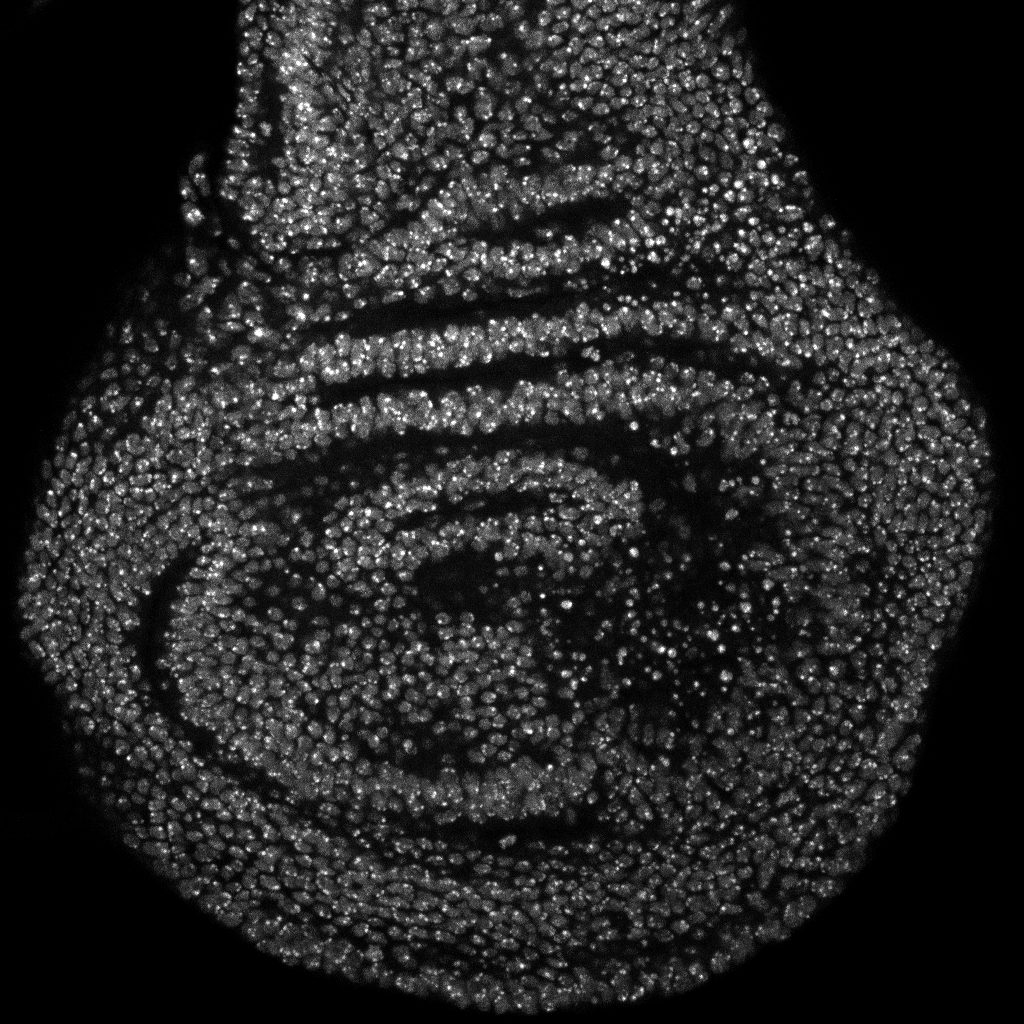

Supplement: Supplementary file 8 — EV Figures Source Data [file 44318_2024_155_MOESM8_ESM.zip › Fig. EV1/Fig EV1 I/nuclei egrweak rnai traf1 new.tif]

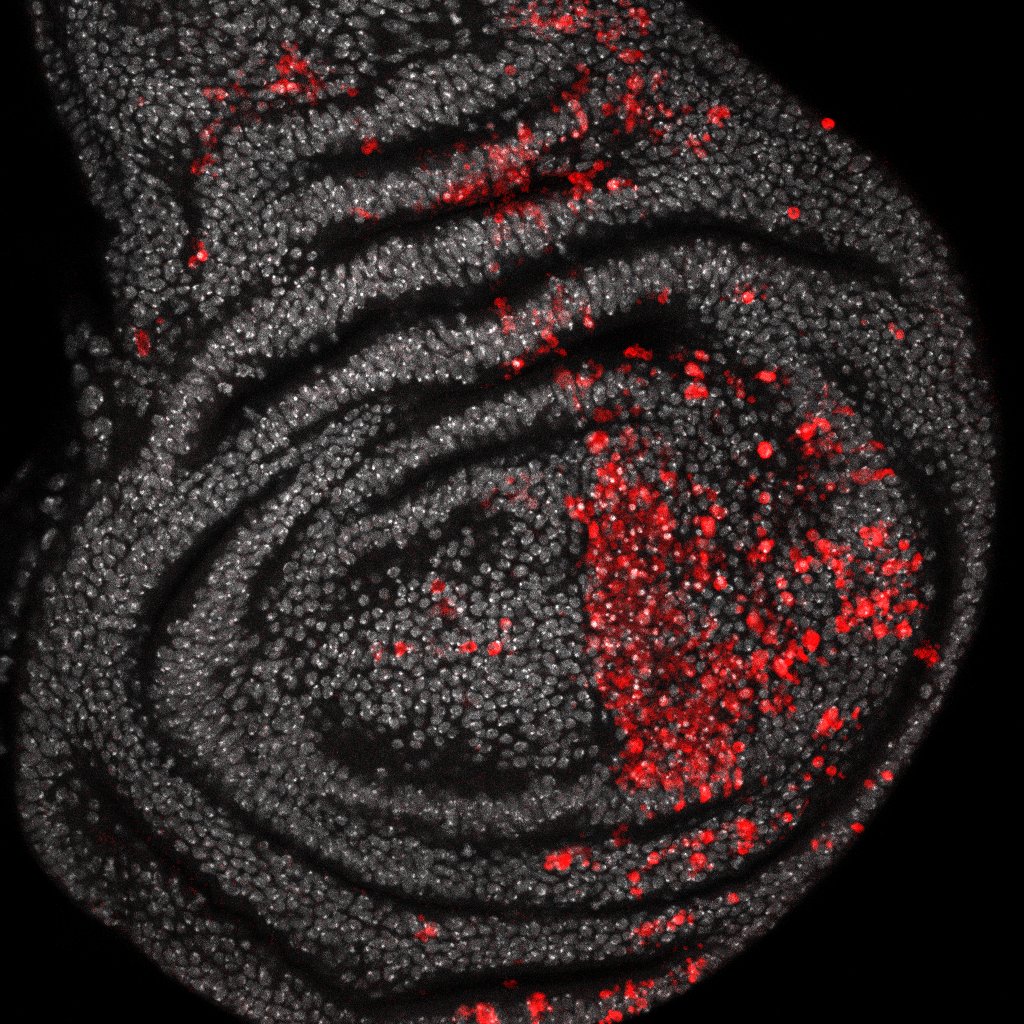

Supplement: Supplementary file 8 — EV Figures Source Data [file 44318_2024_155_MOESM8_ESM.zip › Fig. EV1/Fig EV1 J/Composite rnai ask1 .jpg]

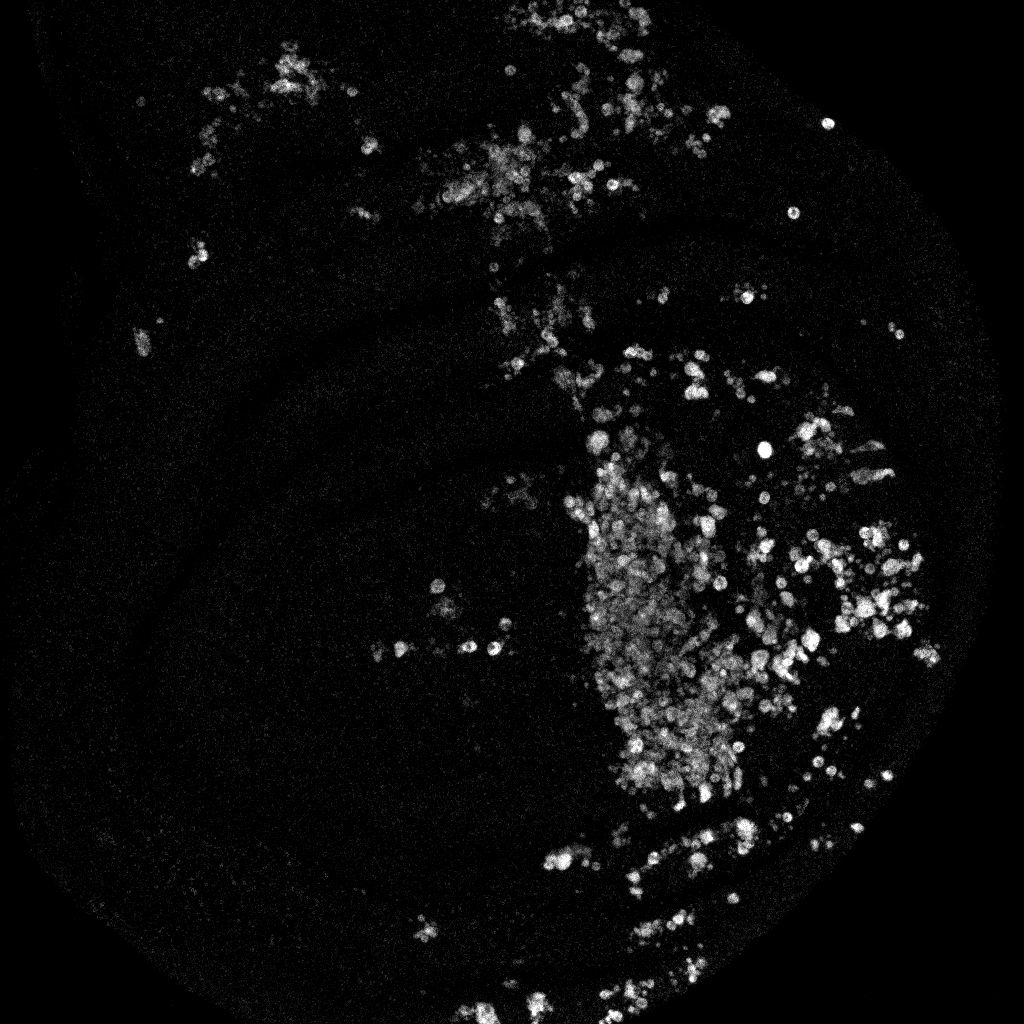

Supplement: Supplementary file 8 — EV Figures Source Data [file 44318_2024_155_MOESM8_ESM.zip › Fig. EV1/Fig EV1 J/dcp1.tif]

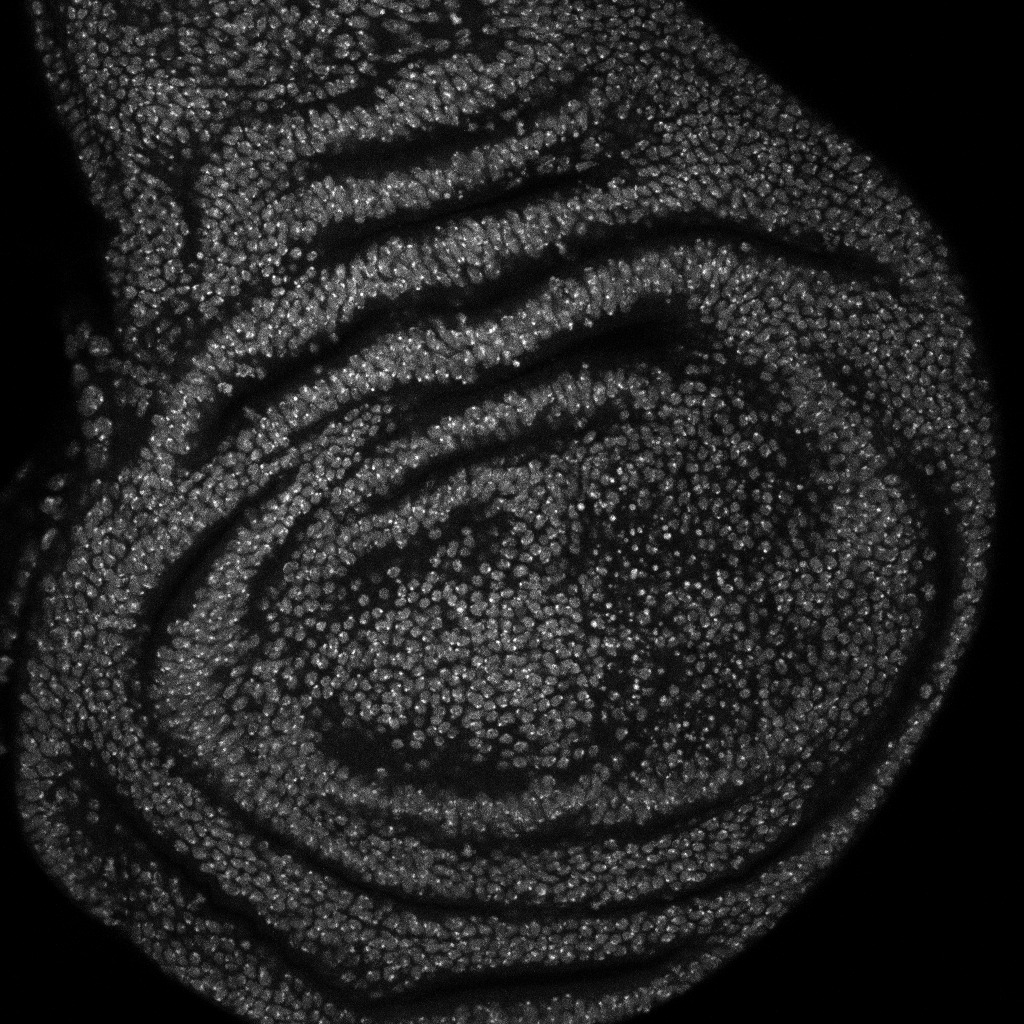

Supplement: Supplementary file 8 — EV Figures Source Data [file 44318_2024_155_MOESM8_ESM.zip › Fig. EV1/Fig EV1 J/nuclei rnai ask1 .tif]

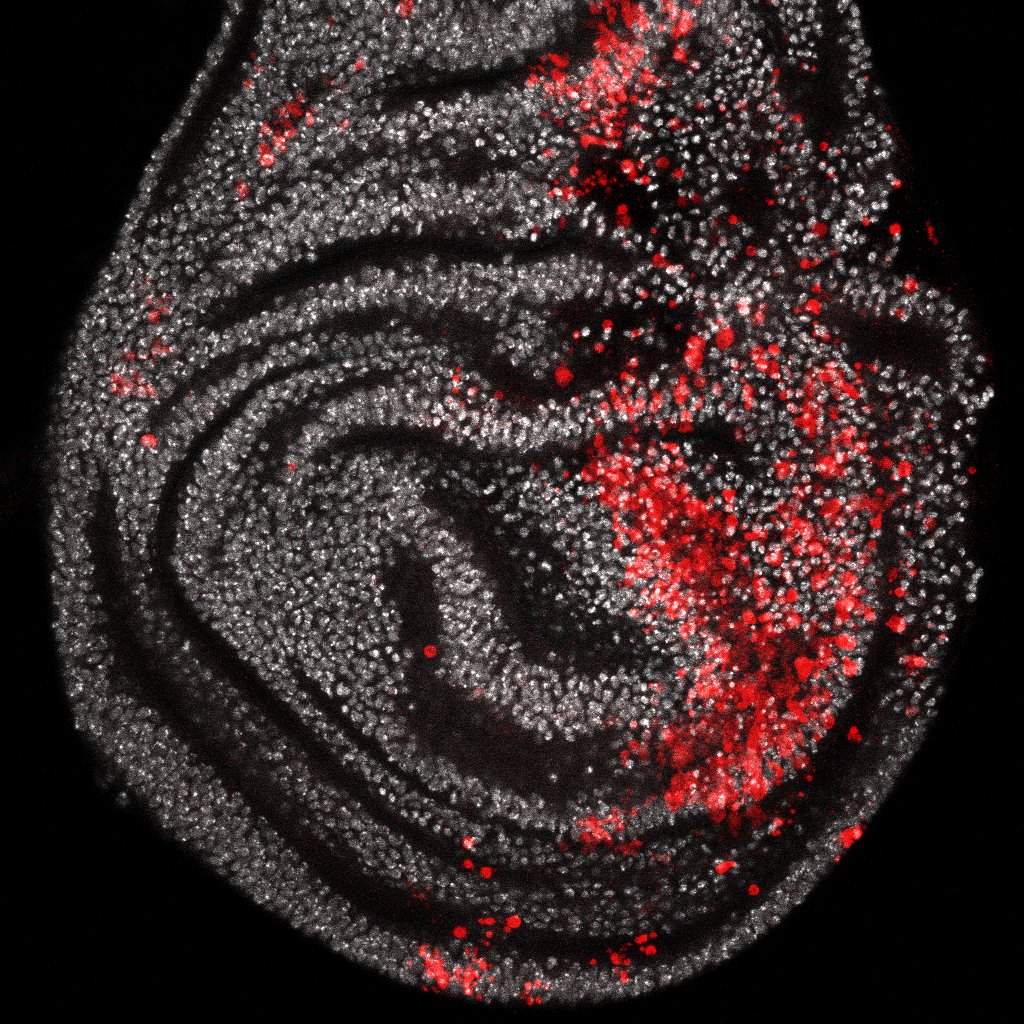

Supplement: Supplementary file 8 — EV Figures Source Data [file 44318_2024_155_MOESM8_ESM.zip › Fig. EV2/FIG EV2 A/Composite enai np38.jpg]

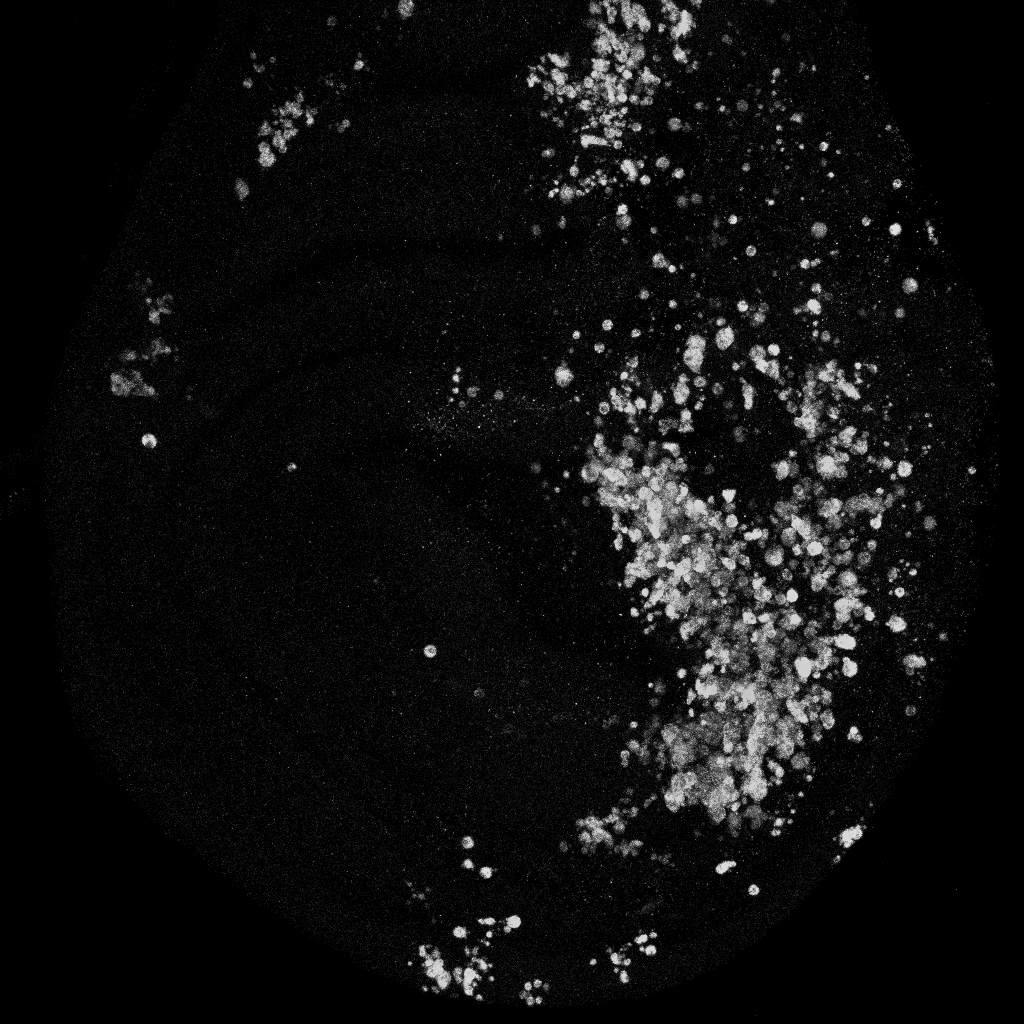

Supplement: Supplementary file 8 — EV Figures Source Data [file 44318_2024_155_MOESM8_ESM.zip › Fig. EV2/FIG EV2 A/dcp1 rnai p38.tif]

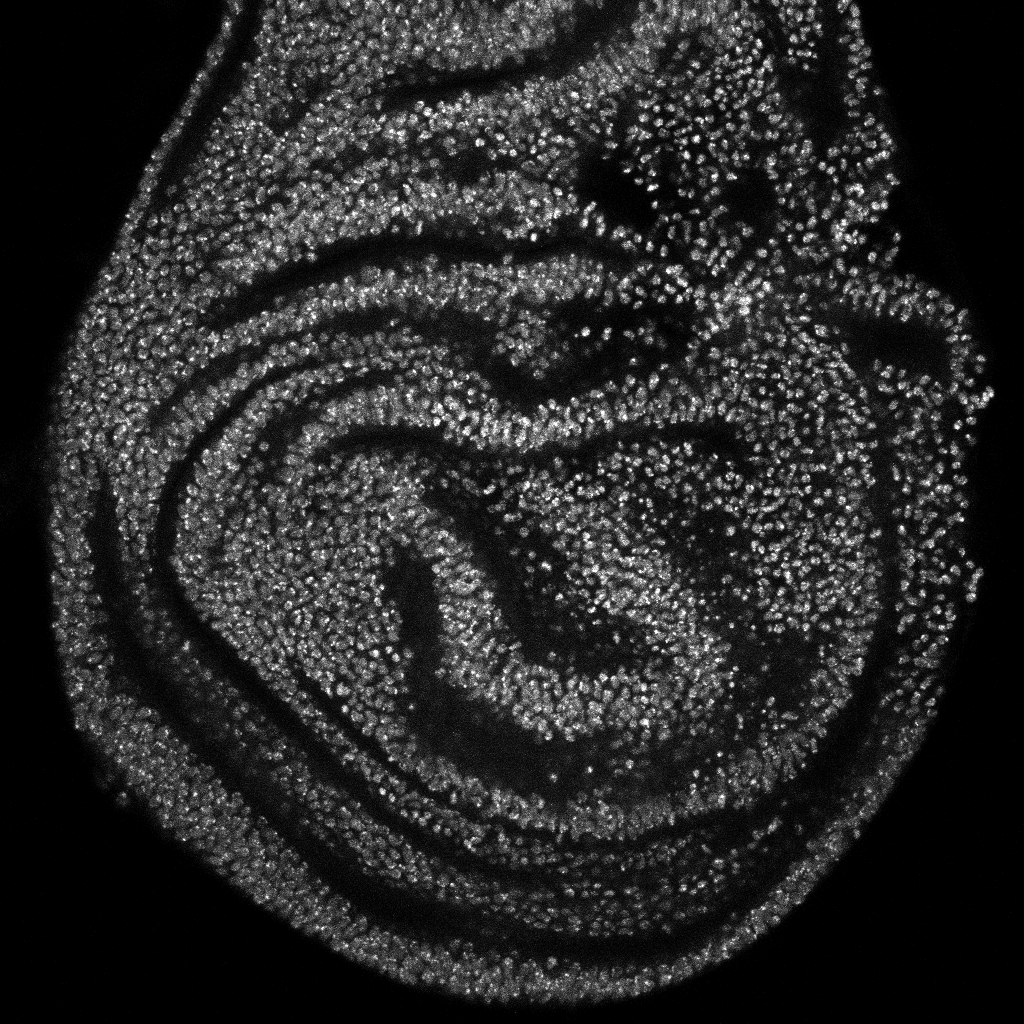

Supplement: Supplementary file 8 — EV Figures Source Data [file 44318_2024_155_MOESM8_ESM.zip › Fig. EV2/FIG EV2 A/nuclei rnai p38.tif]

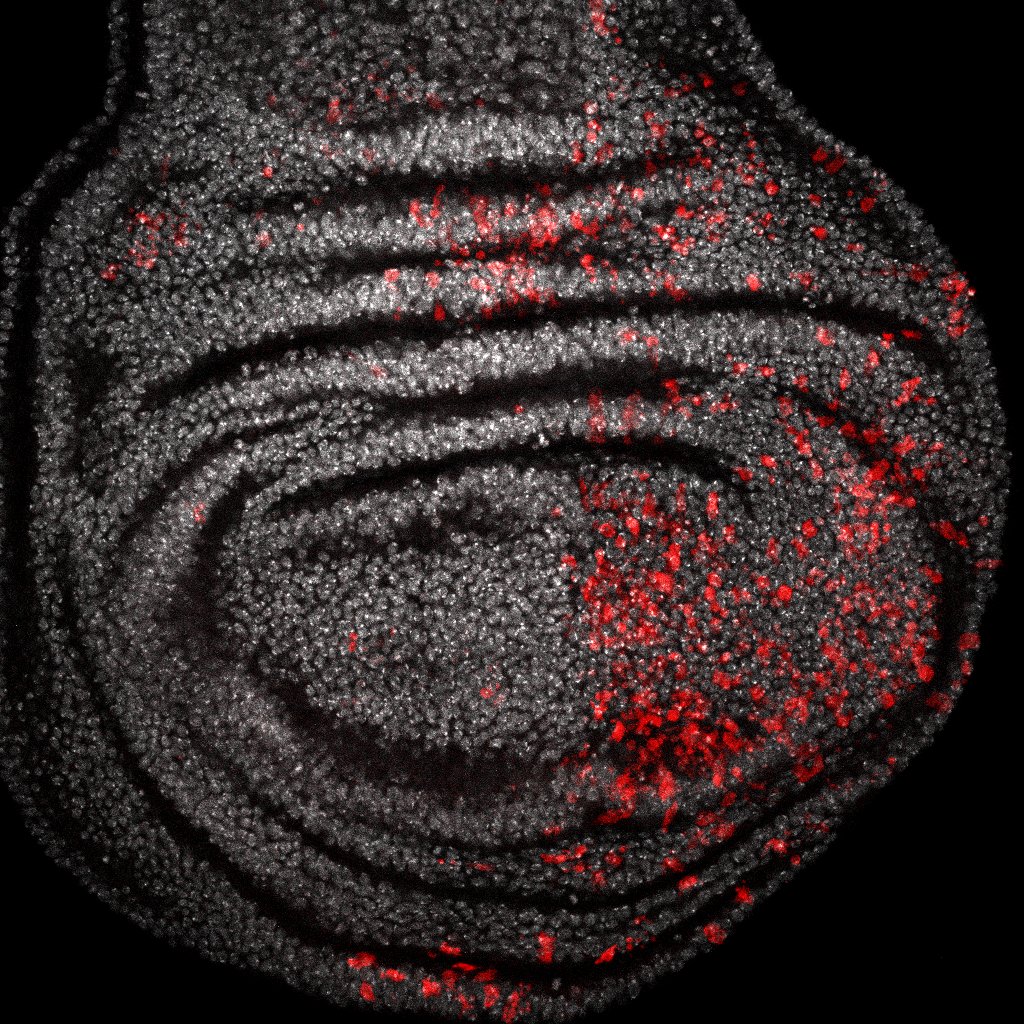

Supplement: Supplementary file 8 — EV Figures Source Data [file 44318_2024_155_MOESM8_ESM.zip › Fig. EV2/FIG EV2 B/Composite lic wt.jpg]

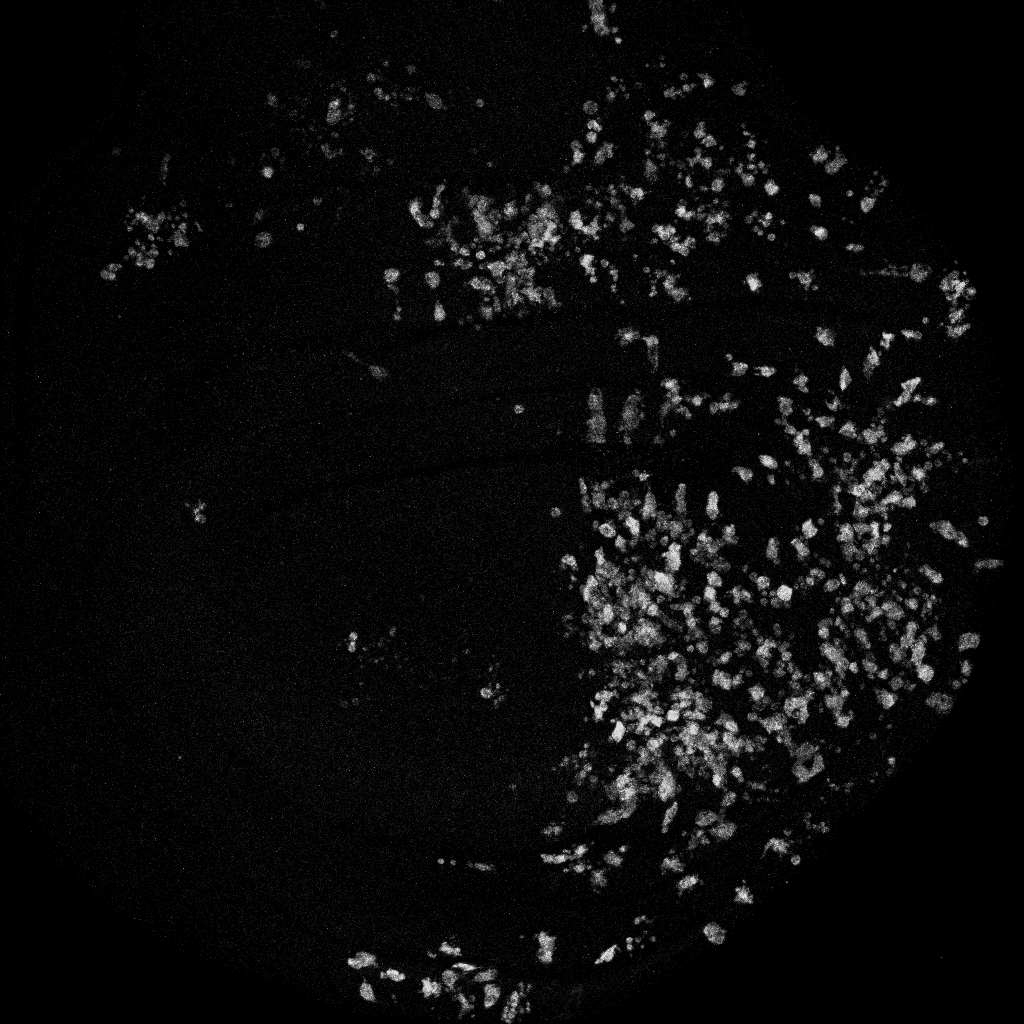

Supplement: Supplementary file 8 — EV Figures Source Data [file 44318_2024_155_MOESM8_ESM.zip › Fig. EV2/FIG EV2 B/dcp1 lic wt.tif]

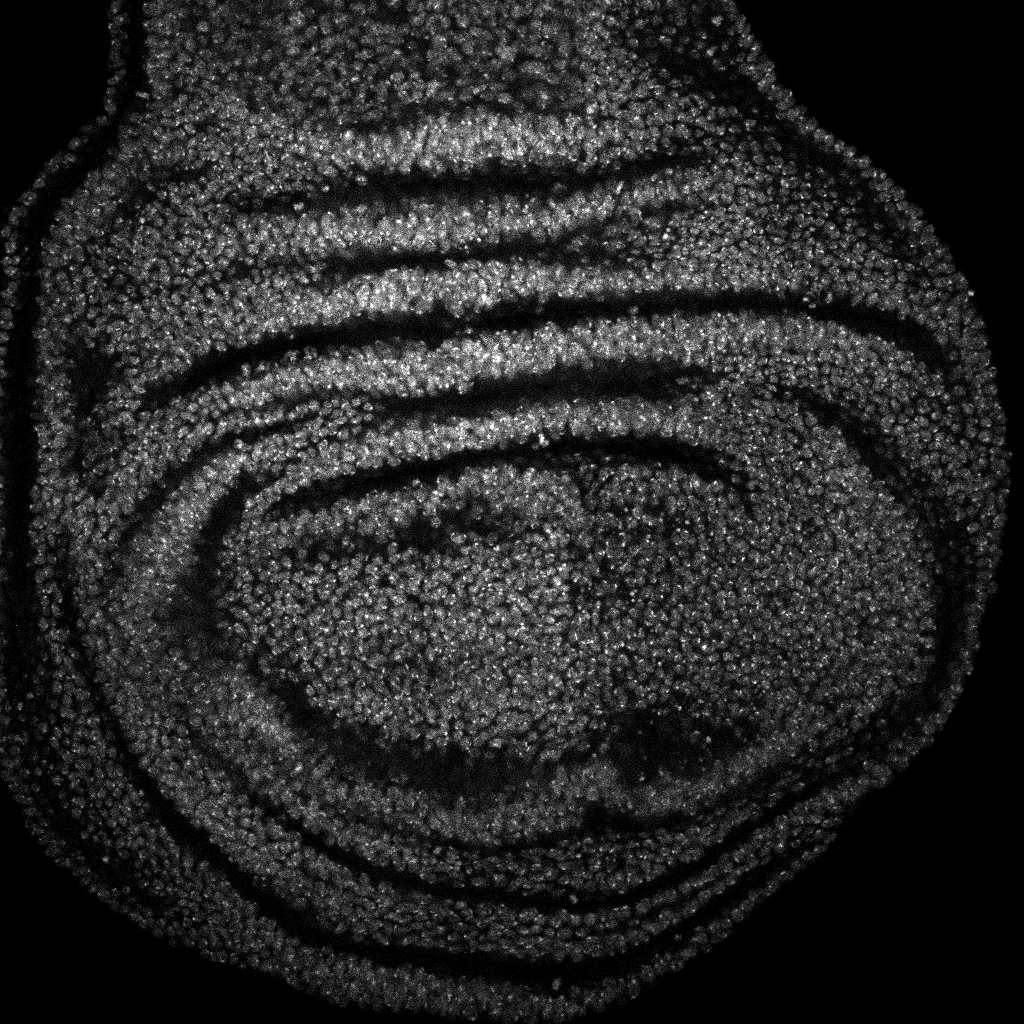

Supplement: Supplementary file 8 — EV Figures Source Data [file 44318_2024_155_MOESM8_ESM.zip › Fig. EV2/FIG EV2 B/nuclei lic wt.tif]
