# Supplementary material for: Reactive oxygen species activate the Drosophila TNF receptor Wengen for damage-induced regeneration
Source: EMBO J. 2024 Jul 17;43(17):3604–26. doi: 10.1038/s44318-024-00155-9 (PMC11377715; doi:10.1038/s44318-024-00155-9)
Supplement: Supplementary file 9 — Expanded View Figures [file 44318_2024_155_MOESM9_ESM.pdf]

Expanded View Figures

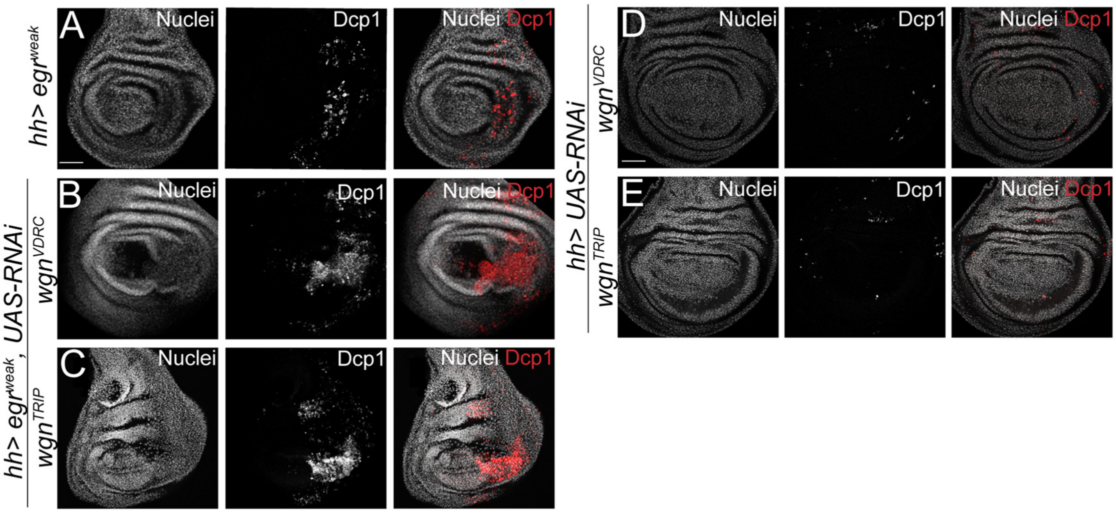

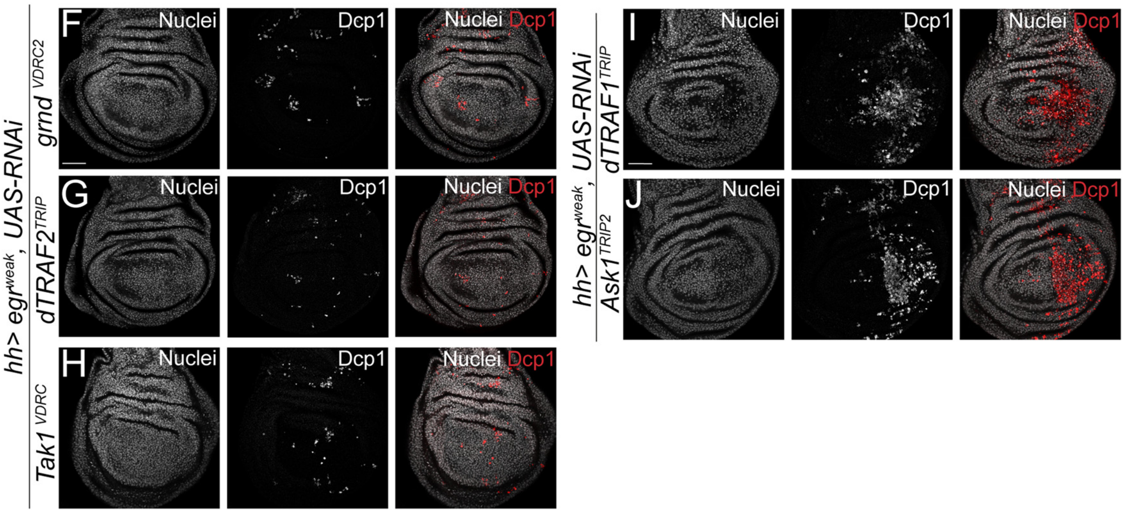

Figure EV1. Dcp1-positive cells in different RNAi strains expressed under *hh-Gal4*.

(**A**) Control *egr^{weak}* overexpression only. (**B**) *egr^{weak}* and *wgn RNAi* from VDRC. (**C**) *egr^{weak}* overexpression and *wgn RNAi*, TRiP strain. (**D**) Control expression of *wgn RNAi*, VDRC alone. (**E**) Control expression of *wgn RNAi*, VDRC alone. (**F**) *egr^{weak}* and *grnd RNAi*, TRiP. (**G**) *egr^{weak}* and *sTRAF2 RNAi*, TRiP. (**H**) *egr^{weak}* and *Tak1 RNAi*, VDRC. (**I**) *egr^{weak}* and *dTRAF1 RNAi*, TRiP. (**J**) *egr^{weak}* and *Ask1 RNAi*, VDRC. TP3 was used to stain nuclei. Scale bar: 50 µm. Source data are available online for this figure.

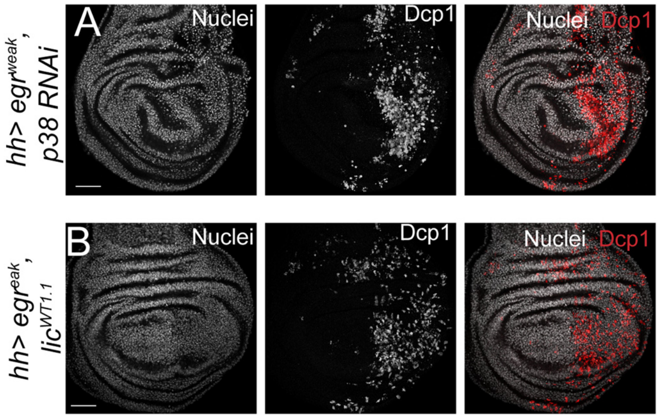

Figure EV2. Reduction or increase of p38 activity results in an increase of apoptosis in *egr^{weak}* tissue.

(**A**) *UAS-p38-RNAi* and *UAS egr^{weak}* transgenes were co-expressed under *hh-Gal4*. (**B**) *UAS-lic$^{WT1.1}$* and *UAS egr^{weak}* transgenes were co-expressed under *hh-Gal4*. Nuclei staining was done with TO-PRO-3 and apoptosis with the caspase Dcp-1. Scale bar: 50 µm. Source data are available online for this figure.

 © The Author(s)

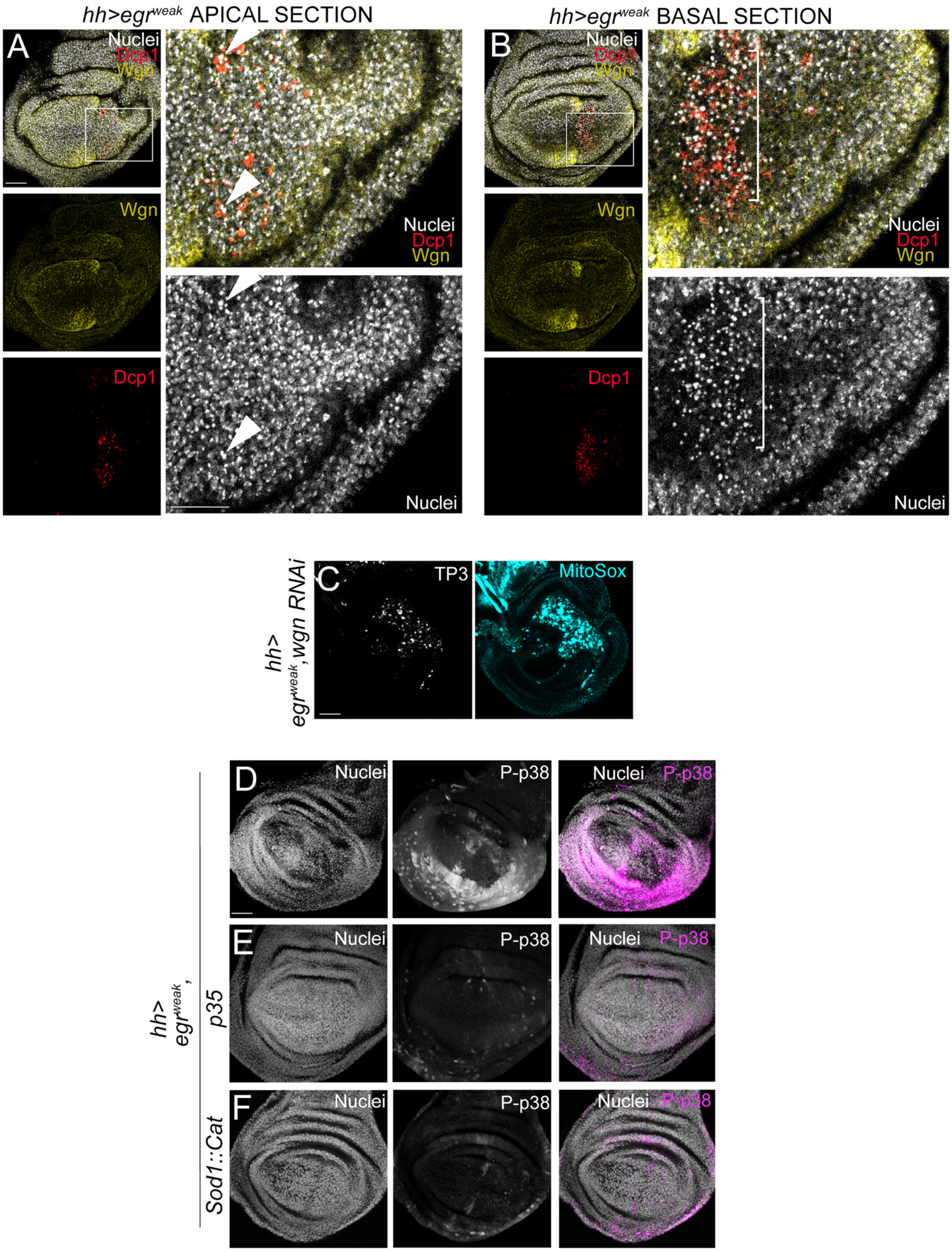

Figure EV3. p38 activation depends on ROS generated by apoptotic *egr* cells.

(**A**, **B**) *Hh-Gal4 UAS-egr*^{weak} wing disc to show pyknotic nuclei positive for the cleaved caspase Dcp1 in apical-basal polarity. (**A**) Plane close to the apical side of the epithelium with a high magnification of the square zone. Note that pyknotic nuclei coincide with Dcp1 cells (e.g., arrows). (**B**) Plane close to the basal side of the epithelium with a high magnification of the square zone. Note that pyknotic nuclei coincide with Dcp1 cells (e.g., bracket), and that the concentration of apoptotic cells is more abundant in the basal area. (**C**) Discs stained ex vivo after co-expression of *egr*^{weak} and *wgn RNAi*. Cell death (TP3), ROS of mitochondrial origin (MitoSOX). (**D–F**) Phosphorylation of p38 after ectopic expression of *egr*^{weak}. (**D**) The transgene *egr*^{weak} was activated in the posterior compartment (*hh-Gal4*); in this optical section there is an accumulation of pyknotic nuclei, typical of apoptotic cells in the center of the posterior wing pouch. Phospho-p38 is found in cells surrounding the apoptotic cells. (**E**) The expression of the apoptosis inhibitor *p35*, and (**F**) the ROS scavengers *Sod1::Cat* concomitantly with *egr*^{weak} result in a strong reduction of phospho-p38. Scale bar: 50 µm.

© The Author(s)

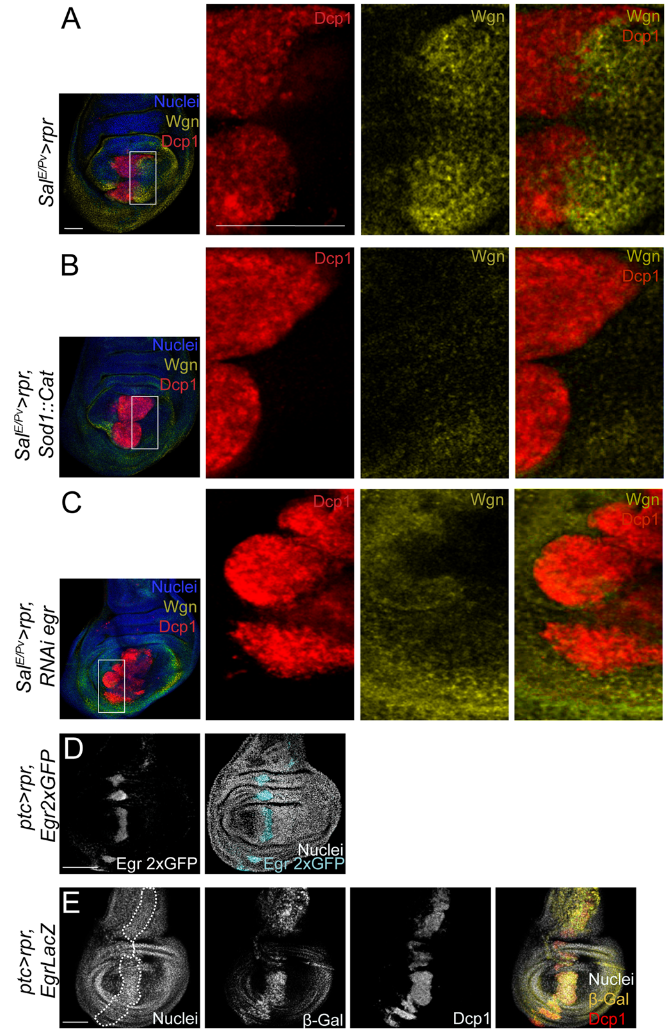

Figure EV4. Wgn response to apoptotic ROS occurs independently of Egr/TNFα.

(**A–C**) High magnification of the interphase between dying cells and responding tissue corresponding to Fig. 5. (**A**) Anti-Wgn and Dcp1 of disc with genetically induced apoptosis using *$sal^{E/Pv}$>rpr*. (**B**) Anti-Wgn and Dcp1 of *$sal^{E/Pv}$>rpr, Sod1:Cat*. (**C**) Anti-Wgn and Dcp1 of *$sal^{E/Pv}$>rpr, egrRNAi* ($n = 19$). (**D**, **E**) Apoptosis genetically induced in *ptc>rpr* in two different Egr reporter backgrounds; (**D**) *Egr2xGFP* reporter and (**E**) *EgrLacZ* detected by anti-ß-Gal antibody. The yellow zone in the merged image shows co-localization of ß-Gal-positive cells and Dcp1-positive cells (dead cells). The dotted lines outline pyknotic nuclei of apoptotic cells. TP3 was used to stain the nuclei. Scale bar: 50 µm.

 © The Author(s)

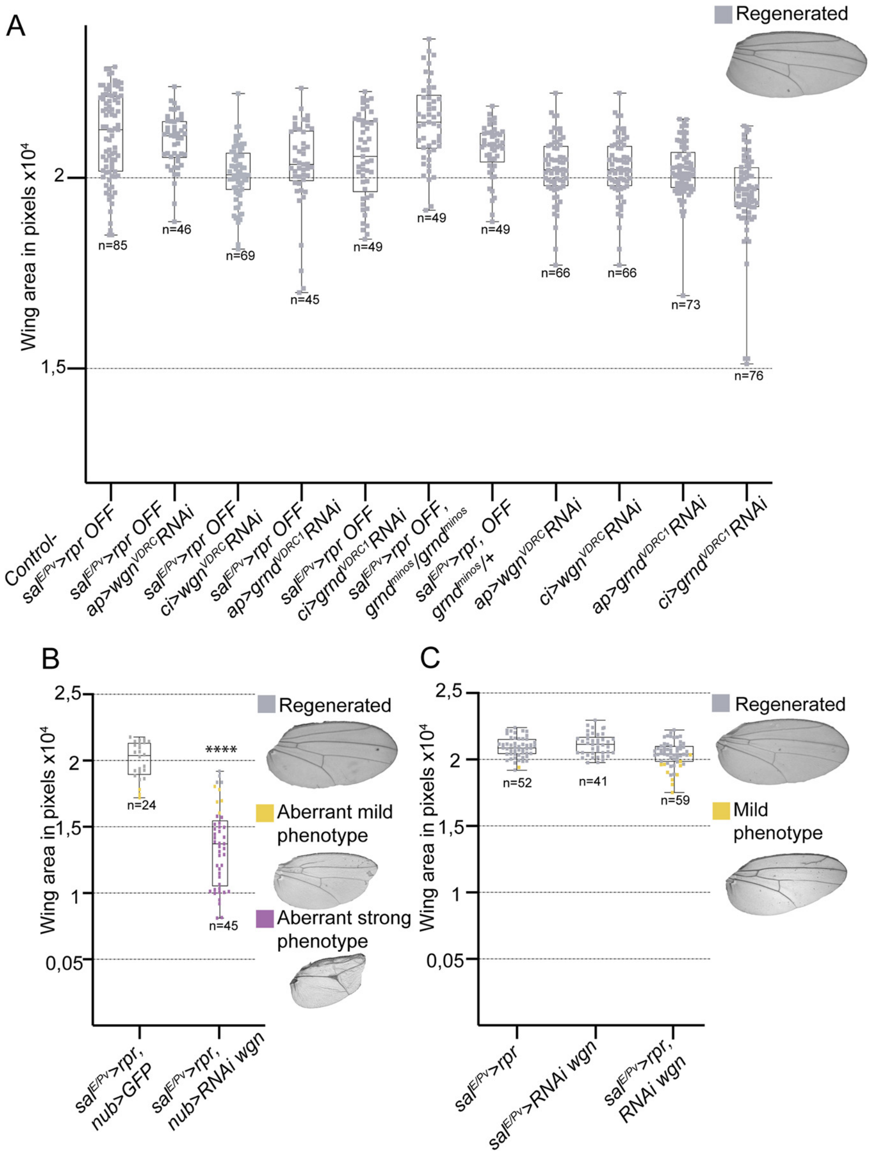

Figure EV5. Control for regeneration assay.

(**A**) Box plot: Y-axis shows the average area in pixels of adult wings obtained from controls kept at 17 °C, with no cell death induction (*$sal^{E/Pv}$-LHG,LexO-rpr* OFF) and no expression of the transgenes. It also shows the average area in pixels from adult wings after the sole expression of the *RNAi* or mutant background (genotypes indicated in the X-axis). Each dot represents one wing: wild-type pattern (gray). One-way ANOVA test was used for multiple comparisons between all groups. (**B**) Regeneration assay. Box plot: Y-axis shows the average area in pixels of adult wings obtained after apoptosis in the *$sal^{E/Pv}$* region and the *wgn RNAi* in the *nub* zone (as in Fig. 6F, G). Each dot represents one wing; wild-type pattern/regenerated (gray), mild aberrant phenotype (yellow), and strong aberrant phenotype (purple). T-Student test was used for comparison of the means between the two groups: ****$p < 0.0001$. (**C**) Regeneration assay. Box plot: Y-axis shows the average area in pixels of adult wings obtained after apoptosis in *$sal^{E/Pv}$>rpr*, in *$sal^{E/Pv}$> wgn RNAi* and in *$sal^{E/Pv}$>rpr + wgn RNAi*. Each dot represents one wing; wild-type pattern/regenerated (gray), mild aberrant phenotype (yellow), One-way ANOVA test was used for multiple comparisons between all groups. Graphs box plots show maximum–minimum range (whiskers), upper and lower quartiles (open rectangles), and median value (horizontal black line).

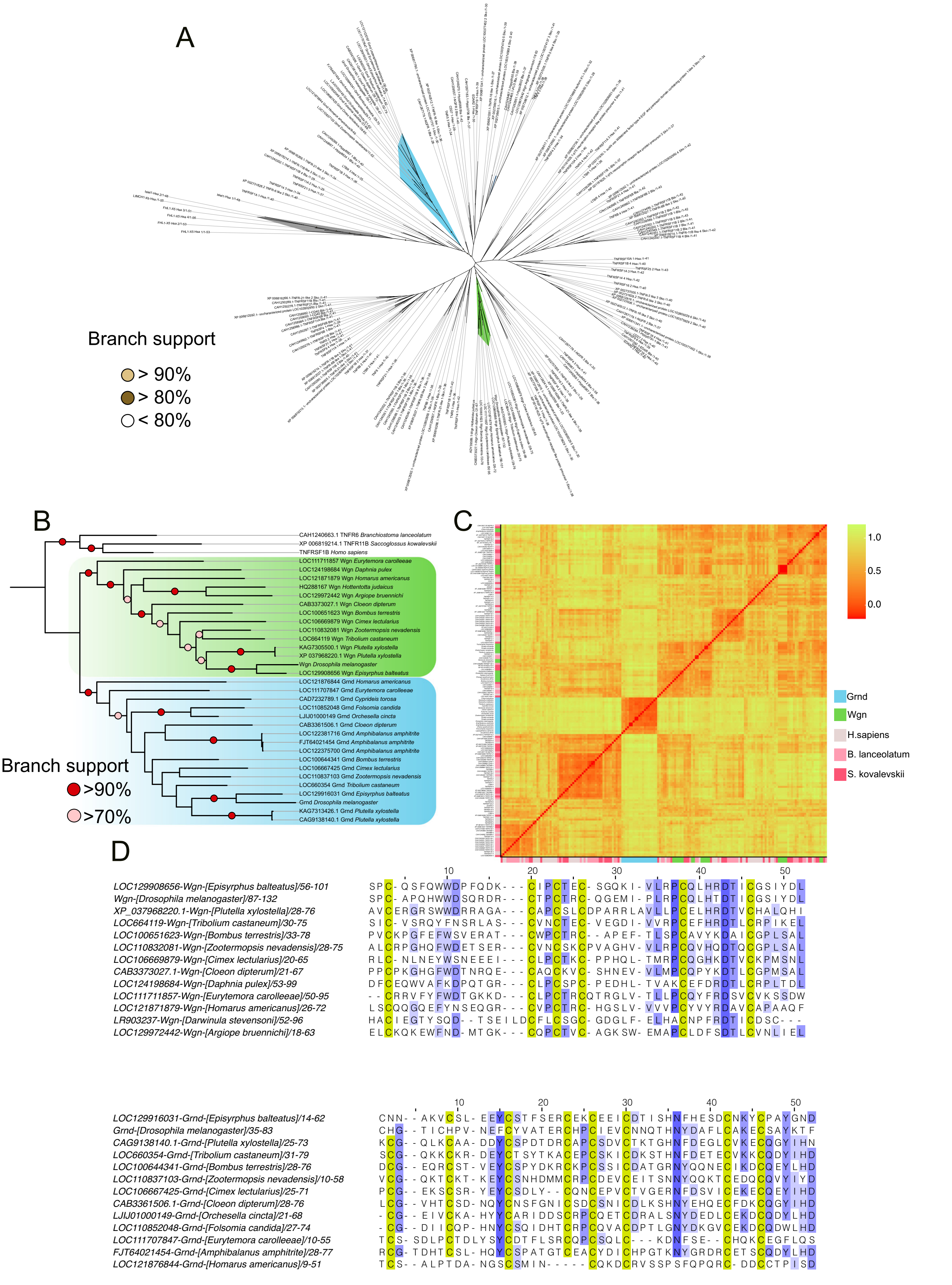
A
Branch support
> 90%
> 80%
< 80%
B
CAH1240663.1 TNFR6 Branchiostoma lanceolatum
XP 006819214.1 TNFR11B Saccoglossus kowalevskii
TNFRSF1B Homo sapiens
LOC111711857 Wgn Eurytemora carolleeae
LOC124198684 Wgn Daphnia pulex
LOC121871879 Wgn Homarus americanus
HQ288167 Wgn Hottentotta judaicus
LOC129972442 Wgn Argiope bruennichi
CAB3373027.1 Wgn Cloeon dipterum
LOC100651623 Wgn Bombus terrestris
LOC106669879 Wgn Cimex lectularius
LOC110832081 Wgn Zootermopsis nevadensis
LOC664119 Wgn Tribolium castaneum
KAG7305500.1 Wgn Plutella xylostella
XP 037968220.1 Wgn Plutella xylostella
Wgn Drosophila melanogaster
LOC129908656 Wgn Episyrphus balteatus
LOC121876844 Grnd Homarus americanus
LOC111707847 Grnd Eurytemora carolleeae
CAD7232789.1 Grnd Cyprideis torosa
LOC110852048 Grnd Folsomia candida
LJJ01000149 Grnd Orchesella cincta
CAB3361506.1 Grnd Cloeon dipterum
LOC122381716 Grnd Amphibalanus amphitrite
FJT64021454 Grnd Amphibalanus amphitrite
LOC122375700 Grnd Amphibalanus amphitrite
LOC100644341 Grnd Bombus terrestris
LOC106667425 Grnd Cimex lectularius
LOC110837103 Grnd Zootermopsis nevadensis
LOC660354 Grnd Tribolium castaneum
LOC129916031 Grnd Episyrphus balteatus
Grnd Drosophila melanogaster
KAG7313426.1 Grnd Plutella xylostella
CAG9138140.1 Grnd Plutella xylostella
Branch support
>90%
>70%
C
1.0
0.5
0.0
Grnd
Wgn
H.sapiens
B. lanceolatum
S. kovalevskii
D
10 20 30 40 50
LOC129908656-Wgn-[Episyrphus balteatus]/56-101
Wgn-[Drosophila melanogaster]/87-132
XP_037968220.1-Wgn-[Plutella xylostella]/28-76
LOC664119-Wgn-[Tribolium castaneum]/30-75
LOC100651623-Wgn-[Bombus terrestris]/33-78
LOC110832081-Wgn-[Zootermopsis nevadensis]/28-75
LOC106669879-Wgn-[Cimex lectularius]/20-65
CAB3373027.1-Wgn-[Cloeon dipterum]/21-67
LOC124198684-Wgn-[Daphnia pulex]/53-99
LOC111711857-Wgn-[Eurytemora carolleeae]/50-95
LOC121871879-Wgn-[Homarus americanus]/26-72
LR903237-Wgn-[Darwinula stevensoni]/52-96
LOC129972442-Wgn-[Argiope bruennichi]/18-63
10 20 30 40 50
LOC129916031-Grnd-[Episyrphus balteatus]/14-62
Grnd-[Drosophila melanogaster]/35-83
CAG9138140.1-Grnd-[Plutella xylostella]/25-73
LOC660354-Grnd-[Tribolium castaneum]/31-79
LOC100644341-Grnd-[Bombus terrestris]/28-76
LOC110837103-Grnd-[Zootermopsis nevadensis]/10-58
LOC106667425-Grnd-[Cimex lectularius]/25-71
CAB3361506.1-Grnd-[Cloeon dipterum]/28-76
LJIJ01000149-Grnd-[Orchesella cincta]/21-68
LOC110852048-Grnd-[Folsomia candida]/27-74
LOC111707847-Grnd-[Eurytemora carolleeae]/10-55
FJT64021454-Grnd-[Amphibalanus amphitrite]/28-77
LOC121876844-Grnd-[Homarus americanus]/9-51

© The Author(s)

Figure EV6. Phylogeny and evolutionary conservation of CDRs from Wgn and Grnd.

(**A**) Full version, including all the species names and sequence accession numbers of the ML phylogenetic tree of the CRDs of Wgn, Grnd, and deuterostome TNFRs featured in Fig. 6A. *B. lanceolatum*, *S. kowalevskii*, and *H. sapiens* are abbreviated as Bla, Sko and Hsa, respectively. (**B**) ML phylogenetic tree of all identified arthropod proteins, using full-length protein sequences and three deuterostome TNFRs as outgroups, *B. lanceolatum* TNFR6L (CAH1240663.1), *S. kowalevskii* TNFR11L (XP_006819214.1), and *H. sapiens* TNFR1B. (**C**) Pairwise comparison of Wgn, Grnd, and deuterostome CRDs. (**D**) CRD alignments of some representative Wgn and Grnd proteins.
